# Monosaccharides and Their Derivatives in Carbonaceous Meteorites: A Scenario for Their Synthesis and Onset of Enantiomeric Excesses

**DOI:** 10.3390/life8030036

**Published:** 2018-08-27

**Authors:** George Cooper, Andro C. Rios, Michel Nuevo

**Affiliations:** 1 NASA-Ames Research Center, Moffett Field, CA 94035, USA; michel.nuevo-1@nasa.gov; 2 Blue Marble Space, 1001 4th Ave, Ste 3201, Seattle, WA 98154, USA; 3 Bay Area Environmental Research Institute, NASA Research Park, Moffett Field, CA 94035, USA

**Keywords:** carbonaceous meteorites, interstellar photolysis, enantiomeric excess, monosaccharide, sugars, sugar acid, aldoses, aldonic acid, sugar alcohol, glycerol

## Abstract

Carbonaceous meteorites provide the best glimpse into the solar system’s earliest physical and chemical processes. These ancient objects, ~4.56 billion years old, contain evidence of phenomena ranging from solar system formation to the synthesis of organic compounds by aqueous and (likely) low-temperature photolytic reactions. Collectively, chemical reactions resulted in an insoluble kerogen-like carbon phase and a complex mixture of discrete soluble compounds including amino acids, nucleobases, and monosaccharide (or “sugar”) derivatives. This review presents the documented search for sugars and their derivatives in carbonaceous meteorites. We examine early papers, published in the early 1960s, and note the analytical methods used for meteorite analysis as well as conclusions on the results. We then present the recent finding of sugar derivatives including sugar alcohols and several sugar acids: The latter compounds were found to possess unusual “d” enantiomeric (mirror-image) excesses. After discussions on the possible roles of interstellar grain chemistry and meteorite parent body aqueous activity in the synthesis of sugar derivatives, we present a scenario that suggests that most of Earth’s extraterrestrial sugar alcohols (e.g., glycerol) were synthesized by interstellar irradiation and/or cold grain chemistry and that the early solar disk was the location of the initial enantiomeric excesses in meteoritic sugar derivatives.

## 1. Introduction

A subset of meteorites classified as “carbonaceous” preserves a valuable glimpse of early solar system chemical and physical processes. This is particularly true of the CI, CM, and CR classes of carbonaceous meteorites, which are viewed as the most unaltered (pristine) since their formation [1]. The majority of carbonaceous meteorites are assigned to the “carbonaceous chondrite” class of meteorites, and we will therefore refer to them as CCs. Their constituent pre-solar grains [2] are an intriguing illustration of their antiquity, as they include the oldest known condensates in the solar system, ~4.56-billion-year-old calcium-aluminum inclusions in the Allende and other CCs [3]. Presolar grains also contain evidence of even earlier events such as the origin of the solar system. Isotopes (^60^Fe, etc.) show that supernovae likely had a role in triggering solar system formation [3,4,5,6]. SiC grains, direct condensates from supernova, are found in several CCs such as the Murchison meteorite [5,7]. The analysis of Cr isotopes in several oxide grains from the Orgueil CC also implicates supernovae in solar system formation [3,8].

**Figure 1:**
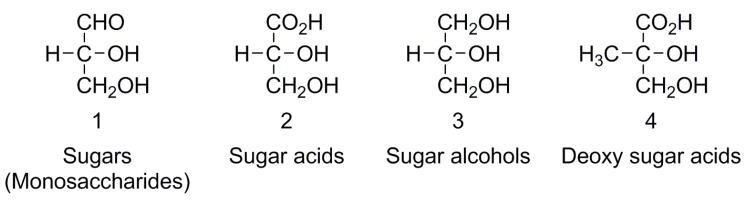
Example structures of polyol classes: (**1**) glyceraldehyde; (**2**) glyceric acid (an aldonic acid); (**3**) glycerol; (**4**) 2-methylglyceric acid.

In addition to supernovae, the presence of multiple other types of stars at (or before) the birth of the solar system [2,3,4,5,6,7] indicates that sufficient ambient radiation was available for the chemical synthesis of organic compounds. For example, analysis of CCs yielded strong evidence of the role of gas-phase photolysis in shaping the properties of certain meteoritic organic sulfur compounds, sulfonates acids (R-SO_3_^−^) (see Section 3.3.3). Characteristics of the organic phase of CCs provide evidence that organic synthetic processes were taking place throughout solar system formation and evolution. This is significant for origin of life research as CCs provide the only window to this prebiotic organic chemistry and can be studied in great detail. The most studied CC, to date, is the Murchison meteorite. The fall of this meteorite (in Murchison, Australia, 1969) was directly observed and it was collected very soon after. This minimized contamination at a time when the lunar program of NASA had developed protocols for the sampling and collection of very clean extraterrestrial samples. In the last few decades, examination of several CCs has revealed that the organic carbon consists of an insoluble “macromolecular” carbon phase, ~50–99% of the total organic carbon [9,10,11,12], as well as multitude of discreet and relatively labile soluble organic compounds [9,10,13,14] that demonstrated an active organic chemistry network in the pre-biological solar system. Identified meteoritic organic compounds are numerous [9] and many are biologically relevant. They include amino acids [9,13,14], polyaromatic hydrocarbons nucleobases [15,16], and early reports of monosaccharides (Section 2.1) and monosaccharide derivatives [17]. Hereafter, we will refer to monosaccharides as simply “sugars”, or refer collectively to monosaccharides and their derivatives as “polyols” (Figure 1). In this article we focus on aldehyde sugars or “aldoses” and their acid and alcohol derivatives. Collectively, these compounds are essential components of DNA, RNA, polysaccharides, and metabolic processes, and are thought to have been necessary for the formation of Earth’s first life forms. Glucose and mannose are familiar examples of polyols in modern biological processes, but glyceric acid (and other sugar acids) also has a critical and ubiquitous role in biology [18]. In this article, we will intermittently use the term “aldonic acid” to refer to the acid form of a sugar in which all carbons are bonded to an OH group. For example, glyceric acid is the aldonic acid form of glyceraldehyde, while 2-methylglyceric acid is a “deoxy” sugar acid (Figure 1).

A note on pristine meteorites: although meteorites generally categorized as “pristine” do show relatively less aqueous and thermal alteration, recent observations have reemphasized the fact that that some do contain evidence of (very localized) aqueous activity. For example, from a study of components of CR2‒3 and CO3.0 carbonaceous meteorites [19], the authors concluded that fine-grained material likely underwent small amounts of pre-accretionary aqueous alteration by melted water ice. The melting could have taken place by multiple mechanisms including shock waves in the solar nebula and heating from ^26^Al inside precursor planetesimals [19]. Another study of the very pristine CR3.0 chondrite MET 00426 [20] illustrated a close spatial association between organic matter and water, and suggested physical and chemical interactions between the two. After an ice-melting event(s), apparently some fraction of the water-soluble component of organic matter was distributed into surrounding cracks and grain boundaries. It was further suggested that this aqueous alteration was of sufficient strength to have transformed soluble compounds into insoluble material. In this article, we will point out how very early aqueous-phase reactions, i.e., before those in meteorite parent bodies (asteroids), may have played a role in shaping the enantiomeric properties of meteoritic organic compounds.

### Enantiomer Properties of Polyols and Their Relevance to the Origin of Life

**Figure 2:**
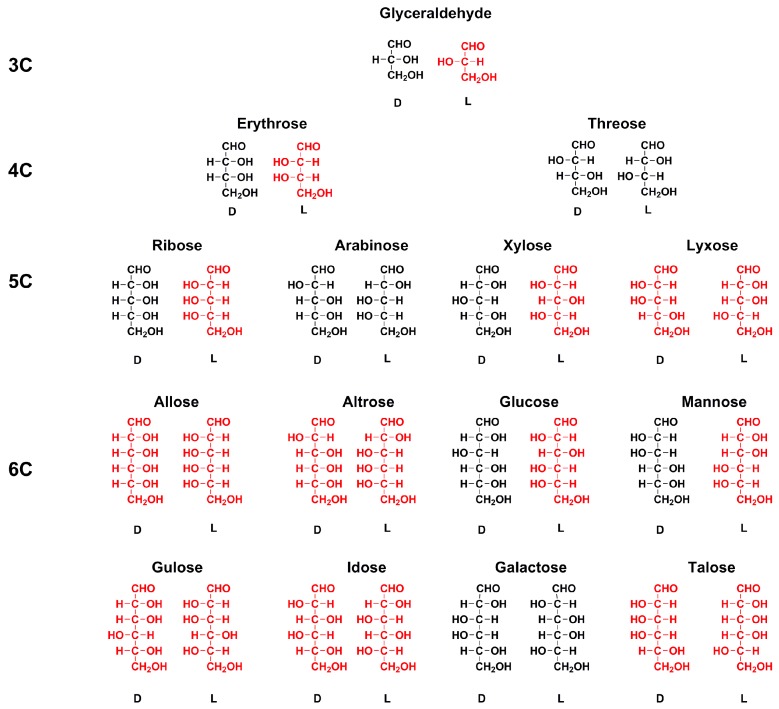
3C–6C Straight-chain sugar (aldose) enantiomers. The biologically more abundant enantiomers are shown in black, and the relatively rare enantiomers are in red. In some cases, the less common enantiomer of a pair is still found in biology, e.g., both enantiomers of arabinose are shown in black. However, the l enantiomer is much more common. The d enantiomers of threose and galactose are more common than the l.

The majority of biological polyols are chiral, i.e., their two mirror images cannot be perfectly superimposed upon each other (where every atom coincides), and each individual member of such a pair is called an enantiomer (usually the “d” or “l”). In this review, compounds that are not chiral will be referred to as “achiral” or “non-chiral”. Figure 2 is an example that highlights the enantiomers of all 3C–6C aldose sugars and denotes whether they are relatively common or rare in biology. A property critical for life is the selective use of just one enantiomer of a pair. The chiral monomers (sugars, amino acids, etc.) of biological polymers such as nucleic acids and proteins are exclusively one of the two possible enantiomers, making the entire polymer “homochiral”. In the case of RNA, only the “right handed” or “d” enantiomer of the sugar ribose is present; in proteins, only “l” enantiomers of amino acids are used. It is still a mystery as to how enantiomer preferences originated in biology [21], however, the phenomenon may have origins in the apparent difficulty of building an abiotic replicating polymer from racemic monomers, i.e., from compounds composed of equal amounts of their two enantiomers (“racemization” is defined as the process of converting one enantiomer into the other; if racemization goes to completion, a 1:1 “racemic” mixture is obtained. It has been shown in one laboratory setting that abiotic syntheses of significantly sized RNA polymers do not proceed in the presence of racemic ribose, as they suffer from “enantiomeric cross-inhibition” [22]. This would imply, if extrapolated back to the origin of life, that a very early and significant enantiomer preference was already in place before the first living cells emerged. In general, there is a scarcity of mechanisms known to be capable of inducing enantiomer asymmetry [23,24] (our term for unequal enantiomer abundances), therefore an important endeavor is to uncover plausible abiotic mechanisms or sources of early enantiomeric enrichments. As will be discussed in Section 2.2, Section 5 and Section 6, all meteoritic aldonic acids and the vast majority of amino acids that contain enantiomeric excesses exhibit the excesses in the same enantiomer as the corresponding biological classes of compounds, the d and l enantiomer, respectively, which is an intriguing similarity to life’s enantiomer selection. Here, enantiomeric excess(es) (***ee***) are determined using the abundances of the individual enantiomers: % ***ee*** = [(l − d)/(l + d)] × 100.

One of the unusual and relevant properties of CCs is that chiral members of one class of compounds will contain ***ee*** while those in other classes can be found as racemic in the same meteorite, for example, ***ee***-containing amino acids [9] and racemic carboxylic acids in the Murchison meteorite [25]. We will address this general question in the discussion of possible locales and origins of polyol ***ee*** (Section 5). While amino acids have been the subject of a long history of analysis [9,26], there are few reports of polyol analysis in meteorites. In the following section we will focus on these publications, which include molecular identifications and the recent analysis of enantiomer ratios of individual polyols. We will then examine polyol synthesis in laboratory simulations under interstellar-type conditions and propose a scenario for producing early solar system enantiomeric excesses. 

## 2. The Reported History of Meteoritic Polyol Research

### 2.1. Early Reports of Meteoritic Sugars

Despite the scarcity of meteoritic polyol research, the essential roles of these compounds in current life and their likely importance at the origin of life warrant a compilation of relevant published literature. Pre-Murchison meteorite studies on the possible presence of polyols in meteorites focused almost exclusively on sugars and no other polyol derivatives. Degens and Bajor [27] presented a short report on the analysis of two meteorites: Bruderheim (an “achondrite”; year of fall, 1960) and Murray (a CC; year of fall, 1950). Sugars were measured at 20 micrograms/gram (i.e., parts per million, or ppm) in Bruderheim and 70 ppm in Murray. However, no analytical details were given, and chirality was not discussed. A follow-up paper that included the original author stated that the original analyses were done with 80% ethanol extraction and acid hydrolysis of the two meteorites [28]. The analytical techniques of the expanded work included thin-layer chromatography, ultraviolet/visible spectroscopy, as well as infrared and nuclear magnetic resonance spectroscopy. Meteorite samples were eight CCs, five non-CC chondrites, and one achondrite. Of the CCs, Murray was the youngest specimen; all others had fallen from 54 to 124 years earlier. Ultraviolet/visible spectroscopy of extracts revealed weak maxima in the region of 225–275 nm, suggesting tentative evidence of sugars, however, peaks were generally very broad or only shoulders and simply suggested a complexity of compounds. The final determination of sugars (qualitative and quantitative) was done by paper chromatography. Detected were the six-carbon sugars glucose and/or mannose, and much smaller amounts of the five-carbon sugars arabinose and xylose. For sugars above trace levels, concentrations ranged from 5 to 26 ppm, with mannose and glucose being the most abundant: The general decrease in order of abundance was mannose, glucose, xylose, and arabinose. No mention was made of enantiomer ratios of individual compounds. In controls and solvents, no sugars or amino acids were found. However, serine was found in relatively large abundances, while non-biological compounds were not reported. As more recent analyses have shown, serine is not a major meteoritic amino acid [29]. Murchison meteorite analyses have nearly all shown that in a series of homologous compounds, rare non-biological members of the series are also prominent and fit within the general pattern of decreasing abundance with increasing carbon number, i.e., such a series represents an abiotic and thermodynamic process [9,10]. The discussion of contamination in the early work (described above) was expanded upon in a contemporaneous review [30] with a general conclusion that contamination was a likely problem in multiple aspects of the analyses. In addition, the major analytical technique, paper chromatography, was described as “an imprecise analytical method” [30]. It is quite possible that the difficulty of meteorite analysis in lieu of contamination from ubiquitous biological sugars (including all of the sugars reported above) discouraged subsequent research in this area for decades.

### 2.2. Recent Reports of Meteoritic Polyols and Their Enantiomer Properties

**Figure 3:**
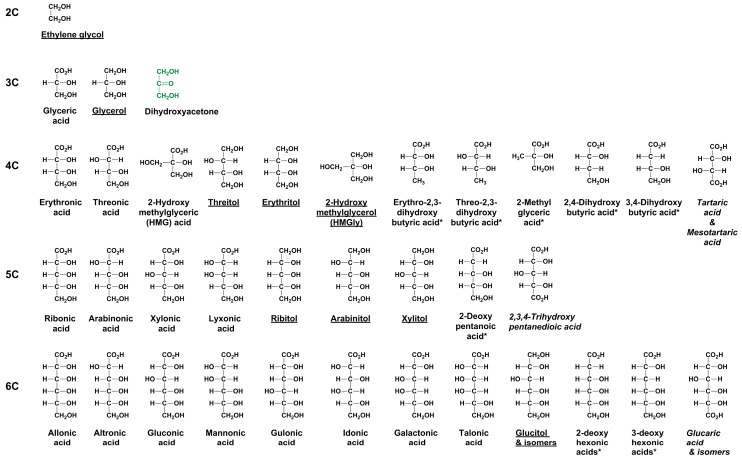
Structures and names of all known polyols identified in carbonaceous meteorites [17,31]. Deoxy sugar acids are marked with (*), sugar alcohols are underlined, and dicarboxy sugar acids are italicized. The only sugar (by definition) is the 3C compound dihydroxyacetone, a ketose that exists in equilibrium with its aldose isomer, glyceraldehyde.

The most recent works on the search for meteoritic polyols reported the finding of one sugar (dihydroxyacetone), several sugar alcohols, sugar mono- and di-acids and deoxy sugar acids [17,31] (Figure 3).

The identifications ranged from the two-carbon (2C) ethylene glycol to several six-carbon (6C) compounds, including a multitude of both biological and non-biological polyols shown in Figure 3. Furthermore, each series of compounds featured a general decrease in abundance with increasing carbon number, as expected for an abiotic synthesis. However, the decrease in abundances from the 3C sugar alcohol (glycerol) and 3C sugar acid (glyceric acid) to their respective 4C homologs was unexpectedly large relative to previously observed homologous series of meteoritic compounds [9,10]. This may have implications for the initial mode of formation of polyols (Section 3).

The origins of the meteoritic polyols were attributed to possible aqueous reactions and/or photolysis of precursors on interstellar grains. Among the rare, non-biological polyols, the detection of branched compounds such as the 4C sugar alcohol 2-hydroxymethylglycerol (HMGly), and the sugar acid 2-hydroxymethylglyceric acid (HMG) (Figure 3) was supporting evidence of abiotic chemistry. In particular, HMG was found to be more abundant than its two relatively common isomers (erythronic acid and threonic acid) in at least one meteorite (GRA 95229) and likely in others [31]. If a portion of these two branched compounds was formed in aqueous reactions [32,33,34,35] a plausible synthetic mechanism is the addition of formaldehyde to glyceraldehyde to give the branched sugar 2-hydroxymethylglyceraldehyde which could then undergo a cross-Cannizzaro reaction to give its corresponding alcohol (HMGly) and acid (HMG) [36]. Of course, any sugars originally present on the meteorite parent body could simply have been oxidized to their corresponding aldonic acids in solution, e.g., ribose → ribonic acid. HMGLy is also a product of low-temperature (~15–80 K) photolysis of interstellar ice analogs (Section 3.2) [37].

**Figure 4:**
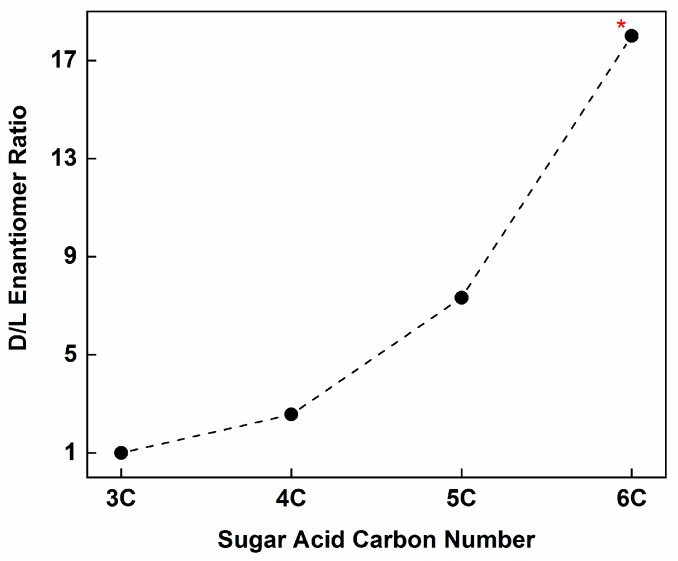
The d/l enantiomeric ratios of meteoritic aldonic acids (Figure 3) increase with carbon number [31]. The asterisk shown in the 6C value is extrapolated based on the d/l trend of 3C → 4C sugar acids as reported in [31].

The enantiomer analysis of polyols in multiple meteorites revealed a surprising pattern of increasing d/l ratios with increasing carbon number (Figure 4). The percent excess of an enantiomer is calculated as shown in Section “Enantiomer Properties of Polyols and Their Relevance to the Origin of Life”. Only d excesses were observed in aldonic acids of ≥4C, while deoxy sugar acids and chiral sugar alcohols were racemic [31].

The smallest (3C) sugar acid, glyceric acid, was found to be approximately racemic in multiple meteorites. This is significant given the ubiquitous nature and high abundance of d-glyceric acid in biology. Large d/l ratios are nearly always observed in soils and even the crust of contaminated meteorites (see Supporting Information (SI) in [31]). Relevant to ideas presented in this article, we note here that since glyceric acid contains only one chiral carbon it would appear to be relatively susceptible to racemization. However, in laboratory experiments simulating plausible meteorite parent body aqueous solutions, no racemization was seen in glyceric acid in the timescale (months) of the experiments (SI in [31]). In addition, the high range of temperatures (~70 °C) in the experiments was sometimes held for extended periods (several weeks), and the pH was often brought to 11 (with Na_2_CO_3_) to hasten any possible racemization. In the astrophysical setting to be presented below, this might imply that, once formed with a particular d/l enantiomer ratio, glyceric acid’s final (observed) enantiomer ratio might reflect the original. But of course, even CC parent bodies may vary from relatively gentle to harsh conditions, and harsh conditions may have racemized any glyceric acid that was initially formed with enantiomeric excess. It is still worth noting that in the recent study of meteoritic polyols, glyceric acid was racemic in a variety of CC [31], at least suggesting the possibility that its enantiomeric ratio reflects early formation processes.

The chiral 4C aldonic acids erythronic and threonic were analyzed in multiple specimens of Murchison and a sample each of GRA 95229 and ALH 85013. These two acids were found to possess an average of 44% d excess in the range of 33–55%. The end-member values came from separate Murchison specimens, and only one measurement of erythronic acid was reported. The authors made note of the fact that one measurement included a 43% d excess of threonic acid in GRA 95229, a meteorite that was demonstrated to be nearly free of observable contamination. However, this raises a question of why certain sugar acids (the aldonic acids) possess enantiomeric excesses while others do not, i.e., the deoxy sugar acids are racemic [31]. This will be addressed below (Section 8) after discussions of possible mechanisms to generate pre-solar enantiomeric excesses.

**Figure 5:**
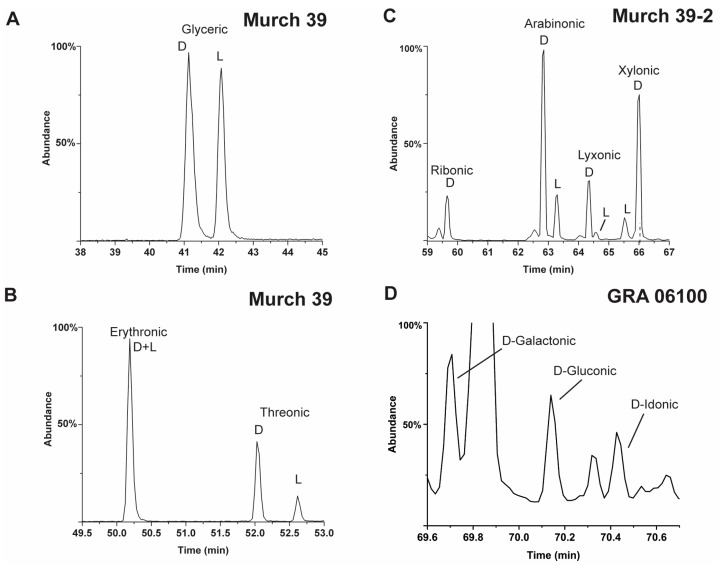
Chromatograms of aldonic acid enantiomer analyses of carbonaceous meteorites [31]. (**A**) 3C (glyceric acid) enantiomers from the Murchison (Murch) meteorite. (**B**) 4C enantiomers. (**C**) 5C enantiomers. (**D**) 6C enantiomers, GRA 06100. No 6C l enantiomers were found. The enantiomers depicted as glyceric acid in (**A**) [31] may actually be from a chiral alkaline or polymer reaction product of glyceric acid (as shown by glyceric acid standards in carbonate solutions). However, other runs of GRA 95229 show that both glyceric acid and possible reaction products are racemic.

The 5C aldonic acids ribonic arabinonic, xylonic, and lyxonic revealed even larger d excesses, both individually and as a group (Figure 5c), than the 4C aldonic acids. In the most clean Murchison sample (M39) ***ee*** ranged from 57% (ribonic acid) to 82% (xylonic acid). Excluding xylonic acid, the ee range narrows considerably: ribonic, 57%; arabinonic, 60%; and lyxonic, 61%. 

6C Sugar acid enantiomer analyses were reported for two meteorites Murchison and GRA 06100. In Murchison, six of the eight possible aldonic acids were identified: Allonic, gluconic, mannonic, gulonic, galactonic, and talonic. Mannonic acid was the most abundant (743 nmol/g) followed by gluconic acid (243 nmol/g). Only d enantiomers were found, which was attributed to the possibility that total abundances in this group were simply too low to observe the l enantiomers. However, despite the overall low abundances, two of the 6C sugar acids, mannonic acid and gluconic acid, were comparable in abundances to the 5C aldonic acids. Therefore, the question: Why were there no observable l enantiomers of the 6C acids, at least for mannonic acid and gluconic acid? One possibility is that, because life’s carbohydrate polymers are composed predominately of the d enantiomer, and the latter two (d) acids are common in biology, they were contaminants and thus the lack of observable meteoritic l enantiomers simply reflects this contamination. On the other hand, it is possible that these 6C acids were indeed indigenous and the lack of l enantiomers simply follows from the observed (indigenous) progression of increasing d excesses, from 4C to 6C (Figure 4) [31]. The authors presented various literature and laboratory experiments in addressing the question of potential contamination. As mentioned, results on six Murchison 6C aldonic acids were shown (excluded were idonic acid and talonic acid). Shown in Figure 5d is a trace of GRA 06100 data from the same work that includes idonic acid, galactonic acid, and gluconic acid, and again, no l enantiomers were observed. Unexpectedly, and in contrast to Murchison, mannonic acid (and the remaining 6C acids) in GRA 06100 is either absent or present in only a trace amount. Due to signs of contamination in GRA 06100, only the 6C sugar acids data was regarded as reliable because six of the eight members (including idonic acid) are biologically rare on Earth. In addition to the above ***ee***-containing discrete compounds, the more abundant organic phase of CC, the macromolecular carbon, also contains a (unknown) source of asymmetry [34]. It was suggested that the increasing ***ee*** (and size) of discrete polyols and the mysterious ***ee*** of the macromolecular carbon are linked by a common synthetic mechanism [31].

It should be stressed that, within each group (4C, 5C, 6C), all of the sugar(aldonic) acids with ***ee*** are diastereomers (i.e., stereoisomers that are not enantiomers). These compounds are relatively difficult (but not impossible) to racemize due to their possession of more than one chiral carbon (SI in [31]) and therefore their d/l ratios are valuable records of an early asymmetrical synthesis in the solar system. However, the 4C deoxy sugar acids 2,3-erythro- and 2,3-threo-dihydroxybutyric acids (Figure 3) are also diastereomers but racemic: Their chiral properties also record early synthesis but apparently one that took place under different conditions from those of the larger aldonic acids.

## 3. The Synthesis of Meteoritic Polyols

In the sections below, we will discuss possible formation pathways (aqueous, photolytic, hydrogen addition) of polyols, without the addition of detailed chemical mechanisms. However, we keep in mind that other factors likely contributed to general synthetic processes, e.g., the reactions of radicals, as supported by the examination of the CC macromolecular carbon phase which revealed that radicals are still trapped constituents, ~4.56 billion years after solar system formation [38]. In addition to more standard reactions, radicals could have played significant roles in potential magnetochiral processes (Section 7).

### 3.1. Meteorite Parent Body (Aqueous) Synthesis of Polyols

Aqueous reactions of formaldehyde and/or glycolaldehyde may be responsible for the presence of several meteoritic polyols such as sugars and/or their derivatives. A likely prebiotic mechanism for their formation, whether on Earth or in extraterrestrial settings, is the “formose reaction” [39,40,41]. This reaction, which is actually a set of reactions, was traditionally described as formaldehyde (CH_2_O) undergoing self-addition in strongly alkaline (high pH) aqueous solution to gradually build a variety of hydroxylated compounds of increasing carbon number. Among the products were said to be glycolaldehyde, ethylene glycol, glyceraldehyde, glycerol, ribose, and other five and six carbon sugars, including glucose, and ketone sugars. However, the formose reaction was also reported not to occur in pure solutions of formaldehyde due to the absence of an enolizable carbon needed to initiate aldol chemistry [42]. Instead, small amounts of organic catalysts, e.g., glycolaldehyde or carbohydrates, were needed [33,42], which created doubt as to the prebiotic plausibility of the formose reaction in the absence of such initiators. Yet, in interstellar environments, formaldehyde is often found with glycolaldehyde [43,44] and many other molecules (for a list of interstellar molecules, including formaldehyde and glycolaldehyde, see [45]). In addition, formaldehyde [46], and glycolaldehyde [47] are constituents of CC and thus a formose-type reaction during aqueous alteration on a meteorite parent body would be chemically plausible.

Historically, the strong base calcium hydroxide (Ca(OH)_2_) is the most common alkaline catalyst in laboratory formose reactions. However, carbonates, i.e., much weaker bases, will also catalyze the reaction but product yields can be substantially lower for any given temperature [41]. Still, as judged from CCs, the asteroid parent bodies likely provided (at least periodically) adequate conditions for these formose-type reactions to occur. In addition to aqueous activity and the presence of formaldehyde and glycolaldehyde, carbonates are relatively abundant in CCs and it is not uncommon to observe up to several percent carbonate in these meteorites [48,49,50]. In addition, pH measurements of CC extracts are often in the ~7–10 range, partly due to carbonates. However, while laboratory formose reactions are generally performed in temperatures ranges of ~50–100 °C, estimates of aqueous (water) alteration temperatures in CC include ~0–50 °C [51] and ~20–71 °C [52]. Measurement of oxygen isotope ratios of water in certain CCs indicate carbonate formation at a “low temperature” range of <150 °C [53]. However, most estimates of aqueous alteration temperature in the most pristine CC favor the lower temperature range, <50 °C. Meteoritic formose-type reactions at <50 °C combined with weaker carbonate catalysts might explain the lower overall relative abundances of each homologous series of polyols, i.e., sugars, sugar alcohols, and sugar acids [17].

Surprisingly, the meteoritic lower mass (2C) alcohol (ethylene glycol), the 3C sugar alcohol (glycerol), and the 3C sugar acid (glyceric acid) were notable for their “over abundance” relative to higher members of their respective homologous series [17]. For example, the 4C sugar alcohols (erythritol and threitol) were estimated to be only ~1% of the abundance of glycerol. A separate study involving organic compounds from a range of CR meteorites of different degrees of aqueous alteration (different petrographic types) also found this strikingly consistent abundance pattern for sugar alcohols [47], as the meteorites ranged from those exhibiting a high degree of (past) aqueous alteration to those that experienced extremely little. From the latter study it can be seen that there is no clear trend across types, with an average abundance of erythritol relative to glycerol and ethylene glycol of ~1% (Table 1; Table S7 in [47]). The ratios and abundances suggest that: (A) more than one mechanism could have contributed to the formation of the lower mass compounds in each series, perhaps through a combination of aqueous, photolytic, and non-photolytic cold-grain chemistry; and (B) aqueous reactions (at least as we know them) were apparently not necessary to form the above-mentioned smaller polyols, i.e., some of the parent bodies of the meteorites had very little evidence of aqueous activity. The studies [47] also reported only trace levels of 5C sugar alcohols (arabinitol, etc.), when they were found at all (Table 1). The constant abundance ratios could also result from the relatively stable (unreactive) nature of the sugar alcohols, i.e., once initially formed at an early pre-aqueous stage they simply persisted through various stages of aqueous alteration.

### 3.2. Interstellar Irradiation Synthesis of Polyols

The foregoing discussions raise the possibility that some fraction of meteoritic polyols were synthesized well before the onset of meteorite parent body aqueous reactions. Meteoriticists have previously concluded that pre-solar material (grains and organic matter) survived the collapse of the solar system’s precursor molecular cloud as well as parent body (asteroid) processes [55].

Although “organic matter” in the above context generally refers to the relatively refractory insoluble carbon phase of meteorites [11,12], low-temperature photolytic reactions in interstellar environments could provide a source for discrete polyol production and therefore an explanation for the anomalous (high) relative abundances of lower mass meteoritic polyols such as ethylene glycol, glycerol, and glyceric acid. In this article, when we refer to laboratory simulations of interstellar (molecular cloud) photolysis we are referring to experiments that generally include a combination of known volatile interstellar molecules (e.g., H_2_O, CO, CO_2_, CH_4_, CH_3_OH, etc.) that condense on the surface of a cold substrate to form “ices” at very low temperatures (~10–80 K). These ices are then subject to irradiation (typically, UV photons) for various periods of times before they are warmed to room temperature under static vacuum, at which time they are recovered for analysis.

Such simulations have added considerable credibility to the hypothesis that irradiation of interstellar grains coated with ices of small precursor molecules was capable of synthesizing prebiotically important organic compounds [56,57,58,59,60,61,62,63]. In the case of polyols, included in the photoproducts are significant amounts of lower mass sugar alcohols and sugar acids [56,64,65,66], smaller amounts of sugars, and the larger molecular weight sugar acids. Initially, questions were raised as to whether the general formation of compounds actually occurred during the warm-up procedure (in preparation for analyses) rather than at the temperature of irradiation. Subsequent experiments have been conducted using laser ablation/ionization mass spectrometry and infrared spectroscopy as in situ analytical techniques that observed the production of compounds in frozen ice mixtures starting at ~5 K [67,68], demonstrating that organic synthesis can occur at extremely low interstellar temperatures.

### 3.3. Formation of Meteoritic Sugar Alcohols: Parent Body (Aqueous) versus Cold Interstellar Synthesis: Are Meteoritic Sugar Alcohols Primarily Products of Cold Interstellar Synthesis?

#### 3.3.1. Sugar Alcohol Production in Standard (Aqueous) Formose Reactions

**Scheme 1:**
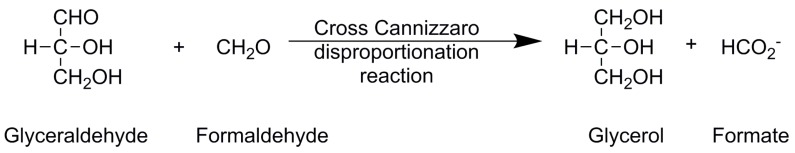
The production of glycerol in the crossed-Cannizzaro reaction [14].

An attempt to understand, at least qualitatively, the relative significance of the two above-mentioned synthetic mechanisms (aqueous versus photolytic) and to discern the location(s) of synthesis of meteoritic polyols, might have implications for theories on the origin of (or lack of) their observed ***ee*** (Section 2.2 and Section 5). An example is glycerol and other sugar alcohols. As mentioned in Section 3.1, conditions in meteorite parent bodies were apparently not optimum for polyol production. Even in laboratory formose reactions, where Ca(OH)_2_ is used as an efficient catalyst for the production of sugars, one report states that “only a small proportion of alditols form” [32] (alditols = sugar alcohols). In fact, even to produce glycerol in formose reactions, “higher temperatures” and “lower pH” were among the necessary conditions [33], as this production is due to the resulting increased rate of the crossed-Cannizzaro reaction. In the formose reaction, the most straightforward way to obtain glycerol would be via the cross-Cannizzaro reaction of glyceraldehyde (Scheme 1):

However, a significant limitation is that glyceraldehyde undergoes this reaction “to only a small extent” [32]. The relative rate of the reaction was thought to be a function of pH and base (catalyst) strength [32,41], as it was found that rates increased with increasing strength of the catalyst. For example, with Ca(OH)_2_ the branched sugar hydroxymethylglyceraldehyde is formed, yet with NaOH (a stronger base), the corresponding sugar alcohol, hydroxymethylglycerol (HMGly, Figure 3) is formed. These observations underscore the point that even with a relatively strong base such as Ca(OH)_2_, it is often difficult to obtain significant yields of sugar alcohols in traditional formose reactions. In fact, the general low abundance of glycerol and higher (≥4C) sugar alcohols in formose-type reactions routinely allow many researchers to convert their complex mixtures of sugars into sugar alcohols (i.e., to create a “reduced formose”) in order to simplify analysis (e.g., [33,36]).

Given the low yields of sugar alcohols using a strong base such as Ca(OH)_2_ in relatively pure laboratory formose solutions, what yields (if any) are to be expected on CC parent bodies? As described in Section 3.1, the most abundant alkaline catalysts available on parent bodies are carbonate salts which are much weaker catalysts for traditional formose-type reactions, especially if they lack significant proportions of calcium ions. In our ongoing formose-type reactions, we have used carbonates (including calcium) as realistic meteoritic catalysts in place of Ca(OH)_2_. We also conducted reactions at temperatures of 2–25 °C for periods of days to months, used formaldehyde or glycolaldehyde as reactants, and these experiments were done with and without added photolysis. Without photolysis, our results show virtually no observable production of either straight-chain or branched sugar alcohols, even when relatively abundant 4C sugars (erythrose and threose) are produced in the glycolaldehyde experiments (one of these experiments might have produced a trace amount of one 4C alcohol). In spite of the above laboratory results, given what could have been long periods of water activity in parent bodies, the production of at least some amount of glycerol seems plausible.

#### 3.3.2. Sugar Alcohol Production by Low-Temperature Photolytic/Grain Surface Reactions

In addition to the laboratory production of multiple polyols using interstellar ice analogs [37,56,64,65,66], recent studies focusing primarily on the production of interstellar glycerol (with isotopically labeled reactants) have offered more evidence of the facile production of this ubiquitous compound. Kaiser et al. [66] used electron irradiation of anhydrous methanol ices to demonstrate the importance of interstellar methanol in glycerol formation. Another study [69], using ices deposited at 15 K, also identifies glycerol and glyceraldehyde. The latter work concluded that, in contrast to more energetic processes (VUV photolysis, cosmic ray, thermal, etc.) at later stages of molecular cloud evolution, the hydrogenation (H addition) of interstellar CO at the relatively early cold, dark cloud stage should also eventually lead to larger sugars and sugar alcohols. These authors also concluded that compounds produced by the hydrogenation of initial CO would be racemic and that any enantiomeric excesses should occur at later stages. Such studies [59,65,66,69] also discuss and demonstrate the likely important role of organic radicals in the production of interstellar organic compounds.

In contrast to the paucity of sugar alcohol production in aqueous-only reactions (Section 3.3.1), relatively large amounts of glycerol are often produced along with lesser amounts of larger (4C–5C) sugar alcohols with added photolysis. The consistent synthesis of sugar alcohols with photolytic formose-type reactions and low-temperature irradiation [37,70], combined with the near lack of production of sugar alcohols in non-photolytic, low-temperature, carbonate-catalyzed formose-like reactions, is qualitatively suggestive of cold grain interstellar mechanisms as the origin of the significant fraction of meteoritic glycerol and other sugar alcohols.

#### 3.3.3. Deoxy Polyol Synthesis: Aqueous Reactions versus Low-Temperature Interstellar Synthesis

**Figure 6:**
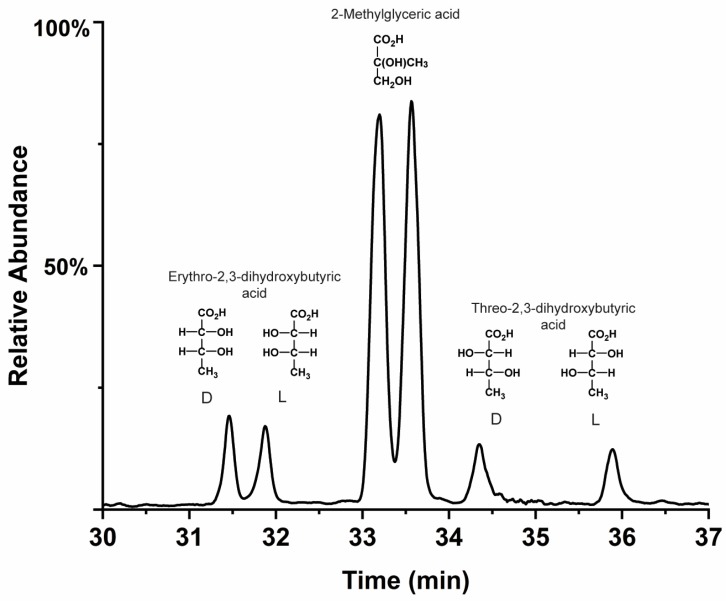
4C Deoxy sugar acids from the Murchison meteorite demonstrating racemic mixtures in this general class of compounds. 2-Methylglyceric acid was also racemic in GRA 95229 in the same extract as non-racemic threonic acid [31]. 2-Methyl-α-amino acids have ***ee*** in these meteorites. Another Murchison deoxy acid, 2,4-dihydroxybutyric acid, is also racemic [31]. Preliminary analysis of Murchison five-carbon homologs of the above compounds, i.e., 5C dihydroxy acids (not shown), are apparently also racemic. These 5C acids were in the same extracts and analyzed with the same methods as the above 4C deoxy and aldonic acids.

As mentioned earlier, the meteoritic deoxy sugar acids, to date, are apparently racemic (e.g., Figure 6).

Deoxy polyols (Figure 3) seem to be even more rare in classical aqueous formose reactions. In contrast, the deoxy polyols 2-methylglyceric acid and 1,3-propanediol were identified in the laboratory low-temperature (80 K) UV photolysis of interstellar ice analogs [36]. The recent report on polyols in meteorites [31] indicated high enrichment of ^13^C in 2-methylglyceric acid and glyceric acid, +82‰ and +60‰, respectively (‰ = parts per thousand), with respect to bulk meteorite and Earth values (the Earth standard for ^13^C/^12^C is Pee Dee Belemnite).This would seem to point to a formation in a very cold interstellar environment, e.g., an interstellar cloud (in contrast, preliminary measurements of the 4C aldonic acids erythronic and threonic revealed lower values, +10‰ each). However, the caveats are that these were single measurements (more are needed for confirmation) and D/H ratios were not analyzed: D/H ratios are also used as markers of formation environment for individual meteoritic organic compounds [9]. In any case, can ^13^C/^12^C and D/H values (‰) of compounds be used in all cases to discern cold pre-solar synthesis (e.g., photolysis) versus warmer parent body aqueous synthesis? For example, while meteoritic organic sulfonates, R-SO_3_^−^ (Section 1), were found to have above-terrestrial bulk values of +6‰ and +600‰ for ^13^C and D/H, respectively, in their hydrocarbon (R) moieties [71], these values were below that of multiple other classes of meteoritic compounds [9] that could well have formed by aqueous processes. However, aqueous processes are ruled out by observations of mass-independent fractionations in the sulfur moieties of the sulfonates [72]. It cannot yet be assumed that one synthetic mechanism assembled the two moieties into the intact sulfonates we now observe.

Despite the paucity of deoxy compounds in formose reactions, the formation of deoxy polyols may be possible under some plausible prebiotic non-photolytic aqueous conditions. For example, the presence of small amounts of 2-deoxyribose was reported from hydrothermal formose-type experiments [73]. The highest yields were reported at 200 °C and 100 bar, with 0.5 M formaldehyde in 0.1 M K_2_HPO_4_. The latter conditions are relatively extreme, however the authors report even smaller amounts under different conditions at 60 °C with Ca(OH)_2_ as the alkaline catalyst [73]. A small number of deoxy sugar acids can also be formed in formose-type or carbohydrate degradation reactions but usually under relatively harsh conditions that include a very strong base (e.g., NaOH) and heating. For example, 2-methylglyceric acid, 2,4-dihydroxybutyric acid, and a few others are obtained by the degradation of carbohydrates (e.g., [17,74]). Although the parent bodies of CC such as Murchison indicate much weaker alkaline conditions (and lower temperatures) than the above, given the time that was available for degradation of (possible) early sugars, the formation of at least some deoxy sugar acids via this mechanism cannot be ruled out. It should be noted, though, that formose reactions performed with and without (sodium) silicate have demonstrated significant preservation of sugars against degradation even in solutions containing the strong base NaOH as the alkaline catalyst [75]. These authors suggested that, due to the ubiquitous nature of silicates, the formose reaction should be considered more prebiotically plausible as the source of early sugars. In the parent bodies of CCs, low temperatures, mild pH silicates, as well as complex-forming cations such as soluble magnesium (relatively abundant) and calcium may have also protected polyols, at least to some degree, from degradation to deoxy and other compounds.

A couple of factors may point to an early indigenous racemic nature of the deoxy sugar acids: (i) although, from a glance at the structure of 2,3-erythro- and 2,3-threo-dihydroxybutyric acids, one could envision a mechanism whereby early ***ee*** could have been lost through racemization, e.g., via isomerization between adjacent (and polar) ‒C‒OH groups, this would still leave the question of why the same type of mechanism did not racemize the 4C erythronic and threonic acids (Figure 5); and (ii) another deoxy sugar acid, 2-methylglyceric acid (Figure 3), has a methyl group on the second carbon and would therefore have been very difficult to racemize if it ever possessed an ***ee***, yet it is racemic while structurally comparable amino acids (i.e., 2-methyl-2-amino) show at least some ***ee*** in the vast majority of CCs [26,76].

## 4. Potential Contributions of Polyols and Precursors from Comets

Comets are generally thought to be the most pristine objects in the solar system [77]. They may provide another window on very early polyol production and chiral characteristics. To what extent are polyols or polyol precursors also found in comets? The European Space Agency’s mission to comet 67P/Churyumov-Gerasimenko (hereafter, 67P) is, to date, the best mission to answer such a question. It features the Rosetta spacecraft and the Philae lander which was equipped with several analytical instruments, including the COSAC (Cometary Sampling and Composition) gas chromatograph‒time of flight mass spectrometer and the Ptolemy ion trap mass spectrometer. These instruments detected several organic molecules [77,78] with abundances or occurrence relevant to the larger polyols in meteorites (asteroids) and other comets. Detected compounds included glycolaldehyde, ethylene glycol, the formaldehyde polymer polyoxymethylene (POM), and a tentative detection of formaldehyde. While a recent article cast doubt on the identification of some of these compounds, including POM and glycolaldehyde [79], a suspected detection of POM was also made by the Giotto mission to comet 1P/Halley [80,81]. Furthermore, laboratory studies have supported these observations, as the irradiation of formaldehyde with VUV photons at 13 K [82] produced several compounds including glycolaldehyde, ethylene glycol, and POM. It was proposed that a radical mechanism could account for the formation of these organics in the laboratory as well as in comet 67P and, possibility, for the presence of glycolaldehyde in star forming regions. 

While it is still unknown the degree to which polyol precursors such as formaldehyde and glycolaldehyde from interstellar syntheses contributed to the observed meteoritic polyols, the extraterrestrial delivery of these precursors by large amounts of comet and asteroid materials could have played a significant role in the activity of polyols on the early Earth. This may be especially significant in the case of a compound such as meteoritic glycerol, where in one measurement its abundance in the Murchison meteorite (~15 ppm) was higher (in general) than that of the most abundant amino acids [17]. Given the ubiquitous and critical role of polyols (glycerol, glyceric acid, etc.) in biology, the ability of glycerol to act as a solvent for organic syntheses [83], and the much higher rate of accretion of carbonaceous asteroidal material early in Earth’s history [84], polyols would likely have been very abundant and available for multiple roles in pre-biological chemistry such as phosphorylation and esterification reactions.

## 5. Possible Mechanisms for Polyol Enantiomeric Excess Production: Laboratory Results

Could the presumed asymmetric influences that lead to the enantio-enrichment of meteoritic sugar acids (Figure 4) also have had an influence on terrestrial prebiotic chemistry and ultimately on the nature of homochirality? Attempts to understand the abiotic origins of these meteoritic excesses might provide some answers. It should be mentioned that several laboratory experiments have produced enantiomeric excesses by means that physically separate pre-synthesized enantiomers, e.g., by differential sublimation, evaporation, melting, etc. However, such compounds are still racemic if the total environment is considered, i.e., if enantiomers in this environment are subsequently re-mixed after short time periods, the formation of homochiral biopolymers might be difficult to achieve due to racemic mixtures [22] (Section “Enantiomer Properties of Polyols and Their Relevance to the Origin of Life”).

Organic synthesis taking place on the surface of grains in interstellar space would have been subjected to ubiquitous forces including magnetism and radiation. A combination of magnetism with unpolarized light has been demonstrated to achieve very small (~10^−4^) but reversible ***ee*** by enantio-selective dissociation of an inorganic complex, i.e., a transition metal (Cr)-oxalate complex [85]. The mechanism for the ***ee*** was attributed to magnetochiral anisotropy (MCA), the influence of magnetism and light upon chirality [85,86]. A requirement for this effect is that the orientations of the light (**k**) and that of the magnetic field (**B**) are parallel (or anti-parallel) to each other. In a recent (unrelated) study, the separation of enantiomers of multiple chiral compounds (monomers and polymers) on magnetized surfaces was achieved [87]. Separation occurred due to the preferential adsorption of enantiomers onto the surface, which depended on the direction (up or down) of their magnetic dipoles. Because the effect is specific for chiral molecules, the authors propose that the method may be useful as a general means of separating chiral from achiral compounds (and also separations within chiral compounds) in mixtures. Independent of this latter work, a means of discriminating chiral from achiral precursors of amino acids and sugar derivatives may have been present at an early stage of solar system formation (Section 6).

In regard to some prebiotically relevant compounds (amino acids), UV radiation in the form of circularly polarized light (UV-CPL) has been demonstrated in laboratory experiments to preferentially degrade slightly more of one enantiomer in a racemic mixture of leucine, depending on the polarization of the light (right or left), leading to small ***ee*** in each case [88].

The above studies were not concerned with the synthesis of compounds during ***ee*** creation, i.e., the target compounds were pre-synthesized. Early attempts to use UV-CPL as the synthetic agent in laboratory simulations of interstellar irradiation included the use of proton irradiation of a gas mixture (CO, NH_3_, H_2_O) followed by UV-CPL irradiation of the resulting soluble residue [89]. This work resulted in the production of small but reversible ***ee*** (depending on CPL direction) of +0.44% and −0.65% in one amino acid, alanine. A slightly earlier attempt of UV-CPL irradiation of an H_2_O:CH_3_OH:NH_3_ ice mixture at ~80 K led to the formation of several amino acids but the measured effect of CPL and subsequent creation of ***ee*** proved inconclusive for alanine and diaminopropanoic acid [90]. A subsequent attempt of a similar experiment resulted in the production of reversible ***ee*** in alanine (^13^C) of up to 1.34% [91]. Finally, the most recent experiments of UV-CPL irradiation of H_2_O:CH_3_OH:NH_3_ ice mixtures led to the synthesis of five amino acids with enantiomeric excesses up to ~2% [92]. Notably, for a given irradiation energy (wavelength) and CPL direction, the sign of ***ee*** was the same for all of the amino acids. It should be noted that in all of the above UV-CPL experiments (including [88]) monochromatic UV light sources were used to achieve the resulting ***ee***, so that experiments employing multiple simultaneous wavelengths (as expected of stellar light sources) would thus be of interest, but not performed to date. Production of ***ee*** is also thought possible from CPL resulting from light scattering of interstellar grains and/or the dichroic extinction of linearly polarized light [93]. Both mechanisms require the presence of non-spherical grains that are aligned in “a common direction”, aided by magnetic fields of specified conformations. CPL from several interstellar sources has been attributed to the above mechanisms [93].

From a plausible prebiotic point of view, the general UV-CPL production of ***ee*** by preferential destruction of one enantiomer (e.g., [88]) raises concerns due to the (common) extent of destruction of both enantiomers in order to achieve the, usually, small ***ee***. For example, in [88], 50–99% of both leucine enantiomers were destroyed during the ***ee*** production. However, if any mechanism is constrained and only capable of, for example, asymmetrically disrupting a chiral reaction intermediate (e.g., a complex) to some extent, this might be sufficient for producing ***ee*** in the final products while allowing relatively high survival rates of the final compounds. An example of this, although from a different mechanism than UV-CPL, is the temporary dissociation of one enantiomer of the chiral Cr-oxalate complex in magnetochiral experiments [85]; if this complex was to then undergo further synthetic reactions, the enantiomer ratios of any chiral products may well be affected.

Could one of the above-mentioned mechanisms (CPL, magnetic, or magnetochiral) account for the observed enantiomeric excesses in some meteoritic sugar acids and not others? As discussed in Section 2.2, in a given meteorite specimen, aldonic acids with ≥4C possess ***ee*** while deoxy sugar acids, glyceric acid (3C) and sugar alcohols are racemic. Achiral photolysis of precursors at low temperatures (interstellar ice analogs) readily produces (racemic) polyols including glyceric acid, deoxy sugar acids, and sugar alcohols [37,56,65,66,68,94]. However, historically, products of the majority of such syntheses are heavily skewed towards the lower mass (2C and 3C) compounds, and often polyols ≥4C are not reported (it should be kept in mind that the present 4C deoxy sugar acids are of lower mass (120 amu) than the present 4C sugar acids (136 amu)). If a synthetic mechanism, or a combination of interstellar mechanisms [94], were responsible for the production of the majority of a group of compounds, then the mechanism’s properties (achiral or asymmetric) could influence the resulting chiral properties of products (e.g., [88,92]).

As discussed in Section 3.3.3, deoxy sugar derivatives are not readily produced in standard aqueous formose reactions, i.e., these derivatives would be less likely products of aqueous reactions inside meteorite parent bodies. While glyceric acid is often seen in laboratory formose reactions, it, and other polyols, were also probably produced in relatively small amounts in parent bodies (Section 3.1). Instead, if certain polyols were as readily produced from interstellar radiation as laboratory simulations suggest then, given the ubiquitous nature of such radiation, a scenario that takes into account the properties of meteoritic polyols (and amino acids), including their ***ee*** and relative abundances, can be constructed.

As proposed by Fedoseev et al. [69], radical addition to a carbon chain could build polyols of increasing size. The proposed mechanism involves the addition of radicals (CH_2_O or CH_2_OH) to the carbonyl end of a given sugar. In contrast, in order to account for the increase in ***ee*** with carbon number of meteoritic sugar acids (Figure 4), here we propose that radical addition to the terminal (and achiral) -CH2OH group of each preceding homolog may have resulted in the unusual and successive ***ee*** increases: terminal addition would have, at the very least, formed the (new) chiral center that by definition identifies an aldose or aldonic acid as either the d or l enantiomer. Such additions could have been to a sugar acid or a parent sugar (the latter would subsequently oxidize to the sugar acid).

## 6. Lessons and Conclusions from Meteoritic Amino Acid Research

As a proxy to possible reactions of polyols on meteorite parent bodies, we must use the much wider knowledge gained from the studies of meteoritic amino acids, in particular their abundance and ***ee*** distribution [26]. It appears that aqueous processes on meteorite parent bodies increased the l-enantiomeric excess of the rare amino acid isovaline [95], CH_3_‒CH_2_‒C(NH_2_)(CH_3_)‒CO_2_H. This amino acid, as with the diastereomeric polyols, is important in the study of meteoritic ***ee*** because it is very difficult to racemize, i.e., it has a methyl group instead of an easily exchangeable H bonded to the carbon (the “alpha”, α, carbon) next to the carboxyl group. To underscore this point, the vast majority of meteoritic α-H amino acids are racemic (see [26] for reported exceptions). Isovaline was found to contain little or no ***ee*** in meteorites that are relatively pristine [47,95,96]: The only report (of small, ~2%) ***ee*** in pristine meteorites is in [47]. In contrast, isovaline’s ***ee*** appears to increase with increasing aqueous alteration of the parent meteorite, up to ~18% ***ee*** [95]. The organic content of the pristine meteorites is thought to generally have formed earlier than that produced in meteorite parent body aqueous processes. It should be noted that isovaline is the only α-methyl meteoritic amino acid, to date, whose d/l ratios are reported from a wide range of meteorites of different petrographic types. For example, two of isovaline’s straight-chained higher homologs, α-methylnorvaline and α-methylnorleucine, also contain ***ee*** but, so far, these are only reported from the aqueously altered Murchison and Murray meteorites [76]. Analysis of these compounds in more meteorites would be interesting. A comparison between isovaline and the deoxy sugar acid 2-methylglyceric acid (Figure 3) shows that while both possess a methyl group on the α carbon and are therefore relatively difficult to racemize, isovaline has ***ee*** while 2-methylglyceric acid is racemic in at least two of the same aqueously altered meteorites (Murchison and GRA 95229; see Figure 6 and [97]). Later (Section 8) we suggest that the formation locale (the precursor molecular cloud) of the significant fraction of 2-methylglyceric acid may be responsible for its racemic nature.

**Scheme 2:**
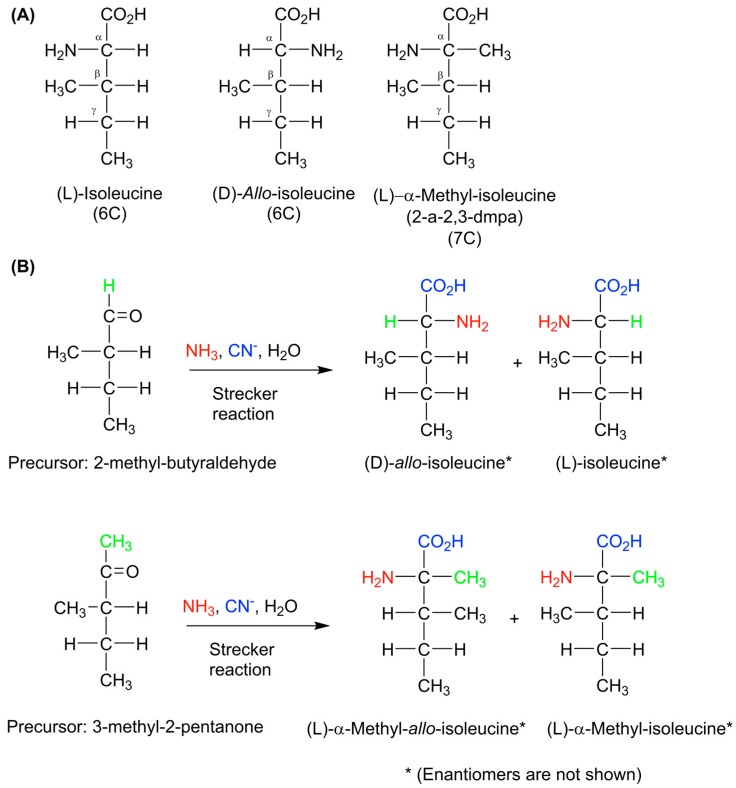
(**A**) The structural relationship between isoleucine, *allo*-isoleucine and α-methyl-isoleucine; (**B**) An illustration of obtaining specific diastereomers in the same Strecker reaction. Each diastereomer has its own enantiomer ratio. From the structures of allo-isoleucine and isoleucine it can be seen that they are not mirror images (enantiomers) of each other. Instead, they are epimers, i.e., stereoisomers that have multiple chiral centers, but only differ from one another by the configuration at one of the chiral centers. It is much easier to epimerize either compound into the other by inverting the stereochemistry at the α carbon because it contains an exchangeable hydrogen. However it is much more difficult to invert both α and β carbons of a given compound to obtain its enantiomer (see text).

Isovaline would have required an achiral precursor (e.g., 2-butanone) for its formation, in the case that its formation took place via the (aqueous) Strecker reaction, which has been suggested as the mechanism of formation for meteoritic amino acids, hydroxy acids [98], and the relatively unusual imino acids [46]. In contrast to isovaline, isoleucine (Scheme 2) and its stereoisomers (6C compounds), which do require a chiral precursor (2-methylbutyraldehyde, Scheme 2) for their formation in aqueous reactions, are shown to contain large ***ee*** (~50–60%) in pristine meteorites [47]. Significantly, these two opposing phenomena, racemic isovaline and large ***ee*** in isoleucine stereoisomers were found together in two of the same pristine meteorites, QUE 99177 and EET 92042 [47,95] (although the meteorites were analyzed in separate studies, and each report focused only on either isovaline or isoleucine stereoisomers). 

Higher homologs (7C) of isoleucine, 2-amino-2,3-dimethylpentanoic acid (2-a-2,3-dmpa) and its diastereomer (Scheme 2, enantiomers not shown) were also found to contain ***ee*** (in Murchison) and, unlike isoleucine, these two compounds have no known natural occurrence on Earth [97]. As with isovaline, 2-a-2,3-dmpa has a methyl group at the α (and β) carbon and is thus very difficult to racemize (for a list and structures of these and all other meteoritic amino acids with ***ee*** see [26]). From a look at the structures (Scheme 2, see also [26,47]), one could picture 2-a-2,3-dmpa as simply isoleucine with a methyl group added to the α carbon. In fact, the common name for 2-a-2,3-dmpa is α-methylisoleucine and its diastereomer is α-methyl-*allo*-isoleucine (Scheme 2). Because of the close structural similarity of these 6C and 7C compounds, it seems difficult to envision separate synthetic mechanisms.

Can a comparison of the structural relationships and enantiomer properties of isoleucine and methylisoleucine shed light on the broader question of the original nature of ***ee*** in at least some meteoritic compounds? From its structure it can be seen that isoleucine has two chiral carbons and therefore so does its diastereomer, *allo*-isoleucine; in both compounds, the α carbon is bonded to H. The ***ee*** of isoleucine was found to be in the l enantiomer and the ***ee*** of *allo*-isoleucine in the d enantiomer [97]. This is understandable if viewed from the perspective of an unbiased aqueous synthesis from the precursor aldehyde, as some proportion of both isoleucine and *allo*-isoleucine will be produced (Scheme 2B). Epimerization (see legend of Scheme 2) will also lead to some equilibrium proportion of the two, as aqueous alteration facilitates the relatively easy exchange of α hydrogens (with water) and leads to inversion of configuration (at the α carbon), thus converting isoleucine into *allo*-isoleucine, and vice versa (Scheme 2). The next carbon in each compound (the “β carbon”) is much more difficult to racemize—due to its position in the chain—even though this carbon is also bonded to a hydrogen. In contrast to isoleucine, the 7-carbon homologs (α-methyl-isoleucine and α-methyl-*allo*-isoleucine) have methyl groups at both the α and β carbon, Scheme 2B. Therefore, once formed, these latter diastereomers will likely preserve a very significant fraction of their original chiral properties at both chiral carbons and, again from an aqueous point of view, should retain their d/l ratios longer than isoleucine and *allo*-isoleucine. Surprisingly, while isoleucine and *allo*-isoleucine had enrichment in their l and d enantiomer, respectively (see above), α-methyl-isoleucine and α-methyl-*allo*-isoleucine both contained l enantiomer excesses [99]. In fact, the authors of that study expected opposite enantiomer enrichments in these diastereomers due to the above arguments of aqueous synthesis (e.g., Scheme 2) (the ***ee*** of α-methyl-isoleucine and α-methyl-*allo*-isoleucine [99] were discovered before those of isoleucine and *allo*-isoleucine [47]).

Here, we propose the possibility that the l-l enantiomer excesses of α-methyl-isoleucine and α-methyl-*allo*-isoleucine could have also been the original case for isoleucine and *allo*-isoleucine (i.e., both were initially l-enriched). But, due to the mentioned relative ease of racemization of the hydrogen atom on the α carbon of isoleucine and *allo*-isoleucine, a portion of l-enriched isoleucine was transformed into d-enriched *allo*-isoleucine (or vice versa), keeping in mind that there was apparently some amount of aqueous activity even in “pristine” meteorites (Section 1), and a possible role of radical chemistry. If epimerization of isoleucine and *allo*-isoleucine were the case, there is the possibility that in the very early solar system there was a driving force that heavily favored the excess production of one enantiomer in similar classes of compounds, e.g., l amino acids and d polyols. With one exception (d-*allo*-isoleucine), all known ***ee*** in meteoritic amino acids are l and all ***ee*** in polyols are d. Because isovaline did not possess a chiral precursor, while isoleucine (6C) and methylisoleucine (7C) (and their diastereomers) did, perhaps the asymmetric synthetic force (CPL, magnetochiral, etc.) acted enantioselectively in the synthesis or destruction of some fraction of chiral precursors at a relatively early stage of solar disk formation. There may be a similarity between the above-mentioned ***ee***-containing and racemic amino acids formed at a very early stage of the solar system (i.e., before parent body aqueous alteration) and the meteoritic aldonic acids. As with racemic isovaline in pristine CC, glyceric acid (racemic) would also not require a chiral precursor, if viewed from a classical aqueous reaction point of view. However, the remaining (***ee***-containing) aldonic acids would have required chiral precursors, similar to the ***ee***-containing diastereomeric amino acids.

It is of interest that both isovaline [100] and isoleucine [101] are also formed in low-temperature UV photolysis experiments. Given isovaline’s near-racemic presence in pristine meteorites, a portion of it could have also been produced along with other racemic compounds via pre-solar disk photo-irradiation (keeping in mind the obvious functional group differences between polyols and amino acids, i.e., an asymmetric reagent (CPL, etc.) may have induced an ***ee*** in one group but not in the other). To account for the observed increase in isovaline’s ***ee*** with aqueous alteration, it was suggested that subsequent aqueous reactions amplified a small initial ***ee*** [95]. The latter two factors, along with the possibility of additional late-stage racemic synthesis, may account for some of the highly variable ***ee*** values observed even within a single meteorite [47]. This reemphasizes the possibility that there were two stages of ***ee*** creation: One was early, evidenced in pristine meteorites, and a later one as seen with isovaline (although analyses of isovaline’s higher homologs across a wider range of meteorite petrographic types are needed to strengthen this point). However, a caveat to this two-stage scenario is that all ***ee*** in meteoritic amino acids (with the above-mentioned *allo*-isoleucine exception) are in the l enantiomer. Likewise, all polyol ***ee*** are in the d enantiomer. Although there are fewer reported polyol measurements relative to amino acids, polyol d excesses do comprise more than ten compounds [31]. What are the chances that all compounds, whether formed early or late, within a single class, would contain the same ***ee***? Was the formation of ***ee*** within, and between, the two classes of meteoritic compounds connected?

## 7. Summary and Conclusions on Meteoritic Amino Acids

The diastereomer meteoritic amino acids isoleucine and *allo*-isoleucine contain large ***ee*** in pristine CC and, from the perspective of a classical Strecker-type (aqueous) formation, “required” chiral precursors for their formation. In contrast, the rare amino acid isovaline required a non-chiral precursor and indeed does not contain ***ee*** (or very little) in the most pristine meteorites. Instead, its ***ee*** appears to be a late-stage (parent body) aqueous event. This raises the possibility that a very early asymmetrical reagent (CPL, magnetochiral, etc.) could have yielded ***ee*** by enantioselectively interacting with, or synthesizing, chiral precursors thus leading to ***ee*** in the corresponding final compounds (amino acids, polyols, etc.). Methylisoleucine (7C) and its diastereomer (each containing two chiral carbons that are extremely difficult to epimerize or racemize) both contain an excess of the L enantiomer whereas isoleucine (6C) and its diastereomer, which also contains two chiral carbons but epimerizes relatively easily because of the α-H, contain an l and d excess, respectively. This leaves open the possibility that the α carbon of isoleucine and/or *allo*-isoleucine epimerized. While true racemization (inversion of configuration at both the α and β carbons) of isoleucine and *allo*-isoleucine is theoretically possible, e.g., *allo*-isoleucine shows a decrease in ***ee*** with increasing aqueous alteration of meteorites (Ref. [47], including SI), however this apparent racemization may also be explained by the additional synthesis of these compounds as racemic mixtures (e.g., in Strecker reactions) The possibility that isoleucine and alloisoleucine underwent epimerization, combined with the fact that all other reported ***ee*** in meteoritic amino acids are in the l enantiomer, suggests the possibility that l enantiomers (of amino acids) could have been the overall “choice” of the solar system’s asymmetrical reagent. The common α-H amino acids may or may not have contained an early (or late) stage l ***ee*** but, as proposed for the α-H carbon of isoleucine, could have undergone racemization.

In the above discussion we mentioned that more analyses (particularly of meteoritic polyols) would be useful. Also, because of the possibility of contamination in meteorites, it should be noted that an ***ee*** of isoleucine (14%) and *allo*-isoleucine (12%) was confirmed by isotope analysis in the relatively clean GRA 95229 meteorite [97] (on the same GC-isotope chromatogram, the enantiomers of the protein amino acid leucine were shown to be racemic). Still, although large ***ee*** were seen in multiple pristine meteorites, isotope analyses of isoleucine (and stereoisomers) have yet to be performed in the above-mentioned pristine meteorites (QUE 99177 and EET 92042) and the possibility of contamination has been raised and answered (see [26]).

## 8. Meteoritic Enantiomeric Excesses: Summarized Facts and Predictions

1. The racemic (and lower mass) meteoritic polyols, deoxy sugar acids, sugar alcohols, and glyceric acid are racemic (or nearly so). These compounds are readily produced by low-temperature photo-irradiation and possibly cold grain chemistry. However, with the possible exception of glyceric acid, they are either not produced or, at best, produced in very low abundances, via weak (i.e., carbonate-catalyzed) non-photolytic formose-type reactions under conditions of carbonaceous meteorite parent bodies.
2. Exposure of interstellar grains and their ice coatings to the ambient radiation UV field [102,103] and cold grain chemistry [69] predated the formation of the solar disk, therefore significant portions of racemic 3C and 4C polyols could have been produced much earlier than their higher mass homologs.
3. There is evidence that aqueous reactions on (the later formed) meteorite parent bodies increased the ***ee*** of isovaline, a rare meteoritic amino acid [95]. On the other hand, observations of pristine meteorites lead to the conclusion that extended aqueous alteration on meteorite parent bodies was not necessary for either the formation or ***ee*** ratios of certain other compounds, i.e., both occurred at an earlier period [47].
4. However, meteoritic sugar acids (i.e., excluding deoxy acids) with higher masses (>3C) carry relatively large ***ee*** which increase with increasing carbon number (Figure 4), as if successive compounds were being constructed from their immediate precursors in a liquid-type medium. Importantly, these sugar acids are found in relatively low amounts (or not reported at all) in the vast majority of past photolysis experiments.
5. If polyols were racemic during an initial synthesis via photo-irradiation or reactions on grains (Item 2) while some compounds contain ***ee*** in pristine meteorites (along with ***ee*** propagation during later-stage aqueous alteration, Item 4), the early solar disk is indicated as the time and locale of ***ee*** production.
6. Magnetochiral effects could be greatly enhanced during the formation of a disk of material, i.e., as the magnetic field intensity in the disk is expected to increase together with increasing density and additive motions of electrically charged ions and grains. In general, the rate and product distribution of chemical reactions involving radicals are known to be affected by magnetic fields [104]. Organic radicals play a significant role in laboratory interstellar ice analog chemistry [59,65,66,69] and such species may have been influenced (directed) by magnetic fields at low disk temperatures. Inorganic (OH) radicals were likely present [11] and may have also interacted with organic species. Although there could be multidirectional magnetic fields in a planetary disk [105], a specific combination of parallel-aligned radiation and magnetic field [85] could theoretically produce enantiomeric excesses. Radicals would play a lesser role in the chemistry of organic compounds, as they (and their precursors) would gradually become shielded from radiation inside of larger objects. The above discussion does not exclude CPL [92] and other mechanisms from ***ee*** production.
7. During aqueous alteration, some of the ***ee***-carrying compounds could have acted as catalysts that induced ***ee*** into other forming compounds (***ee*** amplification). As shown in prebiotically plausible conditions, non-racemic amino acids are capable of acting as catalysts in inducing ***ee*** into sugars during aqueous reactions [106]. However, the singular enantiomer (d) enrichment of meteoritic aldonic acids and nearly complete l enrichment of amino acids would have to be explained by such a mechanism.

## 9. A Chronology for Molecular and Enantiomeric Excess Production of Polyols

**Figure 7:**
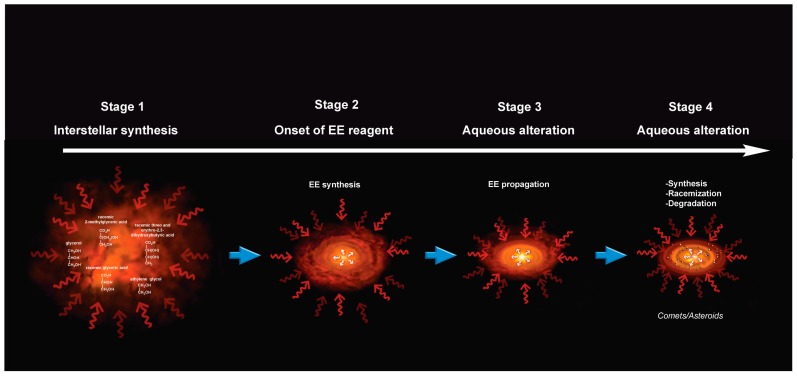
A qualitative depiction of the synthesis and evolution of early solar system polyols including their enantiomeric excesses. A fraction of initially synthesized polyols and other compounds (Stage 1) survive throughout interstellar cloud collapse, T-Tauri, and asteroid/comet stages of planetary disk evolution, while some can be synthesized at multiple stages. The properties of chiral members (see text) are a guide to events at each stage including the timing of ***ee*** production. During Stage 1, racemic deoxy sugar acids and possibly glyceric acid indicate that the formation mechanism was achiral. However, late Stage 4 compounds (e.g., a fraction of isovaline) implicate liquid water in an increase in ***ee***. Between these end members, multiple meteorites with much less evidence of aqueous activity implicate Stage 2 as the beginning of ***ee*** due to the presence of amino acids with relatively large ***ee*** (isoleucine and isomers) and possibly glyceraldehyde (see below). For simplicity, the indicated stages are depicted as single periods. However, research clearly indicates (for one example) that a “Stage 1” could be multiple periods and modes of chemical synthesis, (e.g., [69]). Arrows depict radiation.

The above observations of meteoritic polyols lead to a possible chronology of events in their formation and evolution. Figure 7 gives a simple illustration of the overall scenario. 

**Scenario Stage 1—Molecular Cloud Synthesis of Polyols.** The significant fraction of the observed 2C–4C polyols (especially ethylene glycol, glycerol, glyceric acid and the 4C deoxy sugar acids) were among the very first organic compounds produced in the pre-solar system via ice photochemistry [66,90,91] and/or “non-energetic” synthesis such as CO hydrogenation in ices [69] (athough some could have formed in later aqueous alteration (e.g., Section 3.3.3)). Meteoritic deoxy sugar acids, e.g., 2-methylglyceric acid, erythro- and threo-2,3-dihydroxybutyric acid, etc., are apparently racemic (Figure 6 and [31]) (although small enantiomeric excesses cannot yet be ruled out) therefore the synthetic process(es) was likely achiral. Multiple periods and modes of chemical synthesis within Stage 1 may have occurred: For example, a “CO freeze-out stage” capable of producing such compounds as glycerol and glyceraldehyde, followed by periods of further synthesis by VUV photon and cosmic rays (see [69] and references therein).

**Scenario Stage 2—The Onset of Chiral Influences and Enantiomeric Excess.** At the formation of the early pre-solar disk, an asymmetric reagent(s) became available for the creation of ***ee*** in subsequently produced chiral compounds. For example, the ***ee*** resulting from increased magnetic field strength combined with ambient irradiation, CPL, or CPL from the light scattering of grains and/or dichroic extinction (Section 5). The collapse and concentrating of material (including ions and magnetic fields) from the molecular cloud to a relatively ordered disk would seem to serve the purpose of the above mechanisms. If density of material were a significant factor, then one would expect increased ***ee*** production with decreasing distance to the center of the disk. Although a smaller number of polyols are produced at stage 2, lower mass polyols, including dihydroxyacetone/glyceraldehyde, are known low temperature photo-irradiation products. Once produced in this non-aqueous environment, low temperatures temporarily preserve chiral compounds and their ***ee***. One possible mode of synthesis in the early solar disk, even at low temperatures, is the reaction between reactive ions and radicals (formed by earlier or concurrent irradiation) of close proximity in ices [103]. Despite the general reactivity of radicals, some (usually in aromatic structures) are indefinitely stable. Others, such as di-t-butyl methane radical, C(CH_3_)_3_-CH-C(CH_3_)_3_, are stable in dilute solution, at temperatures (−30 °C in this case) well above interstellar and in the absence of oxygen [107]. Many organic nitroxide radicals, (RR)N-O^•^ are stable under normal conditions; C(CH_3_)_3_-N(O^•^)-C(CH_3_)_3_ is stable to oxygen even above 100 °C [107]. Excursions to higher disk temperatures would increase the molecular mobility of ions and radicals and therefore reaction rates [103]. We note that the melting point range of important precursor molecules such as formaldehyde, acetaldehyde and methanol (unionized at STP) is approximately 149–181 K.

**Scenario Stage 3—The Earliest Stage of Aqueous Alteration.** After the initial period of ***ee*** production, aqueous (or aqueous-like) alteration could have continued the synthesis of small amounts of organic compounds and amplified initial ***ee***. Subsequent syntheses (e.g., of isovaline) could have led to ***ee*** in higher-mass chiral compounds [95] (if their synthetic mechanisms were susceptible) due to interactions with the initial ***ee***-containing compounds. Another possibility, possibly relevant to the question of why the ***ee*** of polyols (and amino acids) are in enantiomers of the same sign (respectively), involves the preserved radicals (or intermediates) from Stage 2. If one driving asymmetric synthetic force is responsible for the single-handed ***ee*** of final products, then perhaps in addition to acting in an enantioselective manner on (or synthesis of) chiral precursors (e.g., precursors to isoleucine, α-methylisoleucine, etc.) at Stage 2, it also biased all chiral intermediate complexes, regardless of whether or not the initial precursors were chiral. For example, one chiral complex in the Strecker synthesis of amino acids is known to be ~C(OH)(H)(CN). Therefore, initially non-chiral precursors (e.g., those that would lead to isovaline, its homologs, etc.) could be biased in the same enantiomer direction as the chiral precursors and their complexes. However, perhaps chiral complexes are not quite as susceptible to an asymmetric force as chiral precursors. There could also be a matter of timing. Because chiral intermediate complexes can of course, only form after their respective precursors, they could have been “left over”, but with an enantiomer bias, from earlier stages of disk evolution. This latter factor, combined with less susceptibility to asymmetric synthetic forces, would imply that the ***ee*** of their eventual products would form later, possibly aided by aqueous reactions, and be less pronounced (have less ***ee***) than the ***ee*** in compounds than began from chiral precursors. On this point, isoleucine (and diastereomer) in pristine meteorites has the largest known ***ee*** of any meteoritic amino acid (~50–60%) whereas, while an ***ee*** of ~18% has been seen in isovaline, much lower values are the norm for this compound (see below). The sugar (aldonic) acids, which also had chiral precursors, also have large ***ee***—and these compounds have not been analyzed in the most pristine meteorites. The slower reaction rates of some precursors versus others may have also played a role in the late generation of ***ee*** in certain compounds. For example, ketones generally have much slower reaction rates compared to aldehydes in substitution reactions such as the Strecker: Isovaline and other (meteoritic) alpha-methyl amino acids are formed from ketone precursors in this reaction (Section 6).

Although the depicted stages are purely qualitative, something akin to a gentle “Stage 3” may be needed to explain the relatively small range (low variation) of ***ee*** observed in meteoritic 4C and 5C sugar acids, respectively [31]. For example, the d ***ee*** of combined 4C acids, erythronic and threonic, clustered around an average of ~43% with deviations of only ~8%. In comparison, much larger deviations are seen in the ***ee*** of meteoritic amino acids: Even within the same meteorite (Murchison) ***ee*** values of 0–15% have been reported for an individual amino acid, isovaline [9]. Another study reported isovaline ***ee*** between 0 to 18.5% depending on CC petrographic type [95]: Other amino acids (stereoisomers of isoleucine) can show wider ***ee*** variations [47]. Such variations seem plausible in compounds that may experience a wide range of physical/chemical conditions on the wet parent body [26] (see Stage 4) combined with their syntheses at multiple stages. Because of this multiple-stage synthesis (which could include episodic racemic synthesis, and racemization), the early (e.g., Stage 1 or 2) chiral nature of compounds such as amino acids may be more difficult to unravel compared to, for example, the sugar derivatives: Note the decline in ***ee*** of isoleucine with increasing aqueous alteration of the meteorite [47]. As described, the synthesis of sugar and derivatives in formose-type reactions in aqueous meteorite parent bodies would seem to be less robust than that of amino acids (produced by Strecker reactions). Therefore, later synthesis would be less likely to mask early polyol characteristics. A lesson from the glimpses we do have of early amino acid enantiomer properties, seen in a limited number of pristine meteorites, indicate a clear preference for chiral precursor compounds in the production of the earliest solar system ***ee***.

**Scenario Stage 4—Full Aqueous Alteration in Meteorite Parent Bodies: Synthesis and Destruction.** This stage features the canonical aqueous alteration in parent bodies. While organic syntheses continued, there is also the destruction and/or racemization of organic compounds [47,95,108]. CC demonstrate a range of degrees of alteration including by oxidation: Oxidizing species such as H_2_O_2_ and ^•^OH likely had significant roles in shaping the properties of CC organic carbon [11]; these processes can preferentially destroy more labile compounds.

To summarize the fate of an individual compound, in the above scenario: Its final ***ee*** is the sum of the 0% ***ee*** produced in synthesis at (racemic) Stage 1 plus the amount of ***ee*** produced in a fresh batch of this same compound at Stages 2 and 3 plus the varying alteration/new synthesis/racemization of its ***ee*** at Stage 4. Partial destruction at Stage 4 would likely not alter enantiomer (d/l) ratios in polyols with multiple chiral carbons (SI in [31]). Therefore, our hypothesis suggests that the bulk of ***ee***-containing sugar acids were made by a relatively homogenous (and benign) process, i.e., during a “Stages 2 and 3”.

### A Note on the Possible Role of Glyceraldehyde in the Enantiomeric Excesses of Meteoritic Polyols

**Scheme 3:**
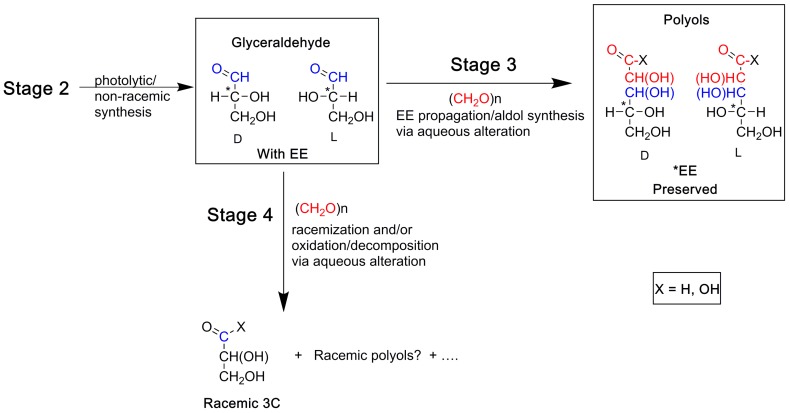
Possible fate of a non-racemic fraction of glyceraldehyde during aqueous alteration. Glyceraldehyde is pictured as reacting with formaldehyde (*n* = 1) or glycolaldehyde (*n* = 2) to produce larger polyols with enantiomeric excess (***ee***). If X = H, 5C sugars are the products; If X = OH, 5C sugar acids are the (oxidation) products. However, glycolaldehyde by itself readily produces sugars (especially 4C sugars), and likely even under the weak formose conditions of meteorite parent bodies (Section 3.1).

Glyceraldehyde, as with other small (2C–3C) compounds presented in this article, can be made by the photolysis of ices (37). Therefore, if following the presented scenario, a portion of it could have been synthesized very early in solar system history (e.g., Stage 1) resulting in a racemic mixture: This fraction would not be a factor in the formation ***ee*** in larger polyols (e.g., the aldonic acids). However, because glyceraldehyde is found in both pristine and water-altered meteorites [47], a portion of it could have acquired ***ee*** at some later stage of solar disk evolution, as apparently occurred with isovaline (Section 6). Although we favor the onset of ***ee*** in the 4C–6C aldonic acids at an early disk stage (a “Stage 2 and 3”), the above non-racemic glyceraldehyde could react with formaldehyde or glycolaldehyde to form (and thus transfer ***ee*** to) larger polyols (Scheme 3). Unreacted glyceraldehyde could subsequently racemize and oxidize, yielding a fraction of the total racemic glyceric acid. However, the ***ee*** initially transferred to larger polyols (such as threonic acid and erythronic acid) would be preserved due to the much higher resistance to racemization of these compounds [31]. It should be noted that persistent small amounts of racemic glyceraldehyde would likely result from the equilibrium with its isomer, dihydroxyacetone, a meteoritic sugar found in low abundance [17] (in fact, glyceraldehyde has now been observed in multiple CCs [47]). The possibility of racemization/alteration/degradation reactions of meteoritic organic compounds in prolonged aqueous environments (e.g., Stage 4, above) is bolstered by the finding that among a group of CR carbonaceous meteorites, glyceraldehyde and other compounds were significantly depleted in the most aqueously altered specimen (GRO 95577) [47]. In general, discrete compounds have the lowest abundances in the most aqueously altered CR and CM meteorites [108]. In spite of this trend, and although glyceraldehyde is more fragile than most polyols, possible destructive processes should be viewed against the reality that glyceraldehyde/dihydroxyacetone are present in meteorites and that polyol ***ee*** [31] and abundance ratios (Table 1) are relatively consistent, indicating their storage and reactions under mild conditions (e.g., Stages 2–3)—at least for some period.

If glyceraldehyde actually never possessed an ***ee*** (or it racemized too quickly) then it was not an asymmetric factor in the production of ***ee*** in larger polyols. This leaves at least two other possibilities for the origin of ***ee*** in the larger (4C–6C) aldonic acids (Figure 5): (i) They were constructed from chiral precursors and acquired their ***ee*** at an early pre-aqueous stage (e.g., Stage 2) due to an asymmetrical force acting preferentially on (or the preferential synthesis of) precursor enantiomers (Section 5); and/or (ii) they acquired their ***ee*** through the effects of neighboring catalytic non-racemic compounds (e.g., [106]) in aqueous solution, as possibly occurred with isovaline (95). The chiral, or non-chiral, nature of their precursors in the latter process may or may not have been the determining factor in the final formation of their ***ee***: Note that ***ee***-containing isovaline likely required a non-chiral precursor (2-butanone) in a Strecker-like aqueous process. This point is significant because the vast majority of biological and known meteoritic amino acids would also have non-chiral Strecker precursors. This leads to the conclusion that such a process could have yielded ***ee*** in a multitude of compounds. However, only those that were difficult to racemize due a relatively stable chiral center, e.g., isovaline, or those that are diastereomers (4C and higher sugar acids, isoleucine isomers, etc.), or compounds that possess both properties, 2-methylisoleucine, etc., are the compounds whose ***ee*** are available to us ~4.5 billion years later.

## 10. Conclusions

Early (~1950s–1960s) meteorite polyol analyses reported the presence of multiple sugars. In all cases, the compounds were identical to those in biology. Although this did not rule out the possibility that some of the reported compounds were indigenous, independent (and contemporaneous) opinion pointed out the possibility of contamination and the need for more definitive analytical methods. More recent analysis of meteoritic polyols revealed a suite of rare and biological compounds including sugar acids and sugar alcohols which appear to be the products of interstellar chemistry (photolysis of ices and cold grain chemistry) and later aqueous processes on larger objects. Although we are unable to know the exact chronology of polyol formation in the early solar system, hypotheses are constrained by the distribution and enantiomeric properties of the measured compounds. Conclusions derived from these properties could be summarized as: (i) Chiral polyols, i.e., those that were synthesized in the pre-solar molecular cloud(s), are racemic; (ii) later (Stage 2), chiral precursor compounds (polyols, aldehydes, ketones, etc.) were differentially influenced by an asymmetric force(s): Chiral compounds, resulting from the latter precursors, contained enantiomeric excesses (***ee***) as evidenced (so far) by certain sugar acids and (a limited number of) amino acids in pristine meteorites; (iii) subsequently, these initial ***ee*** were also catalysts in the creation of new ***ee*** in other (or the same) compounds; (iv) however, the ***ee*** of the latter compounds could also have resulted from storage and slower reaction rates of their precursor compounds and (pre-biased) chiral intermediates; and (v) chiral compounds (whether created at early or late stages) also underwent some degree of racemization and/or chemical alteration. The ***ee***-containing chiral compounds that were resistant to total racemization are the ones we now observe to carry ***ee***. The volume of research on meteoritic polyols is significantly less than that of other compound classes, particularly amino acids, and more analyses are clearly needed. However, early data on enantiomeric excesses in meteoritic polyols, a group that includes biologically rare members, offer hints that extraterrestrial creation of ***ee*** could have played a role in initiating life’s homochirality after seeding Earth.

## Figures and Tables

**Table 1:** Glycerol/erythritol ratios and abundances of 5C sugar alcohols versus the degree of aqueous alteration in carbonaceous meteorites *. No clear trend in ratios and abundances are seen.

Degree of Aqueous Alteration							
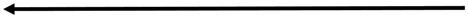							
Meteorite (Petrologic Type)	GRO 95577 (1.3)	PCA 91082 (2.3)	QUE 99177 (2.4)	Murchison (2.5)	EET 92042 (2.5)	MET 00426 (2.6)	MIL 07525 (2.8)
Glycerol/erythritol	89	24	74	100	171	104	70
**5C** Alcohols abundance	tr	tr/nf	nf	tr	tr	tr/nf	tr/nf

* All data from [47] except for Murchison [17]. Meteorites are ordered by petrographic subtype (parentheses), as taken from [1] except for MIL 07525 [54]. Other meteorites with qualitatively large glycerol/erythritol ratios are GRA 95229 and ALH 85013 (calculated data from recent publication [31]). 5C Sugar alcohols refer to ribitol, arabinitol, and xylitol. tr = Trace levels; nf = not found; tr/nf = one of the three alcohols was not found.

## References

[1] Alexander C.M., Howard K.T., Bowden R., Fogel M.L. (2013). The classification of CM and CR chondrites using bulk H, C and N abundances and isotopic compositions. Geochim. Cosmochim. Acta.

[2] Zinner E., Davis A.M. (2014). Presolar grains. Meteorites and Cosmochemical Processes, Volume 1 of Treatise On Geochemistry.

[3] MacPherson G.J., Boss A. (2011). Cosmochemical evidence for astrophysical processes during the formation of our solar system. Proc. Natl. Acad. Sci. USA.

[4] Amari S., Zinner E., Lewis R.S. (1995). Large ^18^O excesses in circumstellar graphite grains from the Murchison meteorite: Indication of a massive-star origin. Astrophys. J. Lett..

[5] Hoppe P., Pignatari M., Kodolányi J., Gröner E., Amari S. (2018). Nanosims isotope studies of rare types of presolar silicon carbide grains from the murchison meteorite: Implications for supernova models and the role of ^14^C. Geochim. Cosmochim. Acta.

[6] Telus M., Huss G.R., Ogliore R.C., Nagashima K., Howard D.L., Newville M.G., Tomkins A.G. (2016). Mobility of iron and nickel at low temperatures: Implications for ^60^Fe–^60^Ni systematics of chondrules from unequilibrated ordinary chondrites. Geochim. Cosmochim. Acta.

[7] Gyngard F., Jadhav M., Nittler L.R., Stroud R.M., Zinner E. (2018). Bonanza: An extremely large dust grain from a supernova. Geochim. Cosmochim. Acta.

[8] Nittler L.R., Alexander C.M., Davidson J., Riebe M.E.I., Stroud R.M., Wang J. (2018). High abundances of presolar grains and ^15^N-rich organic matter in CO3.0 chondrite dominion range 08006. Geochim. Cosmochim. Acta.

[9] Pizzarello S., Cooper G.W., Flynn G.J., Lauretta D., Leshin L.A., McSween H.Y. (2006). The nature and distribution of the organic material in carbonaceous chondrites and interplanetary dust particles. Meteorites and the Early Solar System II.

[10] Sephton M.A. (2002). Organic compounds in carbonaceous meteorites. Nat. Prod. Rep..

[11] Cody G.D., Alexander C.M. (2005). NMR studies of chemical structural variation of insoluble organic matter from different carbonaceous chondrite groups. Geochim. Cosmochim. Acta.

[12] Alexander C.M., Newsome S.D., Fogel M.L., Nittler L.R., Busemann H., Cody G.D. (2010). Deuterium enrichments in chondritic macromolecular material-implications for the origin and evolution of organics, water and asteroids. Geochim. Cosmochim. Acta.

[13] Pizzarello S. (2016). Molecular asymmetry in prebiotic chemistry: An account from meteorites. Life.

[14] Elsila J.E., Charnley S.B., Burton A.S., Glavin D.P., Dworkin J.P. (2012). Compound-specific carbon, nitrogen, and hydrogen isotopic ratios for amino acids in CM and CR chondrites and their use in evaluating potential formation pathways. Meteorit. Planet. Sci..

[15] Stoks P.G., Schwartz A.W. (1982). Basic nitrogen-heterocyclic compounds in the murchison meteorite. Geochim. Cosmochim. Acta.

[16] Callahan M.P., Smith K.E., Cleaves H.J., Ruzicka J., Stern J.C., Glavin D.P., House C.H., Dworkin J.P. (2011). Carbonaceous meteorites contain a wide range of extraterrestrial nucleobases. Proc. Natl. Acad. Sci. USA.

[17] Cooper G., Kimmich N., Belisle W., Sarinana J., Brabham K., Garrel L. (2001). Carbonaceous meteorites as a source of sugar-related organic compounds for the early earth. Nature.

[18] Chiba H., Sasaki R. (1978). Functions, of 2,3-bisphosphoglycerate and its metabolism. Curr. Top. Cell. Regul..

[19] Haenecour P., Floss C., Zega T.J., Croat T.K., Wang A., Jolliff B.L., Carpenter P. (2018). Presolar silicates in the matrix and fine-grained rims around chondrules in primitive CO3.0 chondrites: Evidence for pre-accretionary aqueous alteration of the rims in the solar nebula. Geochim. Cosmochim. Acta.

[20] Le Guillou C., Brearley A. (2014). Relationships between organics, water and early stages of aqueous alteration in the pristine CR3.0 chondrite MET 00426. Geochim. Cosmochim. Acta.

[21] Blackmond D.G. (2011). The origin of biological homochirality. Philos. Trans. R. Soc. B Biol. Sci..

[22] Joyce G.F., Visser G.M., van Boeckel C.A.A., van Boom J.H., Orgel L.E., van Westrenen J. (1984). Chiral selection in poly(C)-directed synthesis of oligo(G). Nature.

[23] David Barron L. (2013). True and false chirality and absolute enantioselection. Rend. Fis. Acc. Lincei.

[24] Meierhenrich U., Brack A., McKay C.P., Horneck G., Stan-Lotter H. (2008). Amino Acids and the Asymmetry of Life.

[25] Aponte J.C., Tarozo R., Alexandre M.R., Alexander C.M., Charnley S.B., Hallmann C., Summons R.E., Huang Y. (2014). Chirality of meteoritic free and iom-derived monocarboxylic acids and implications for prebiotic organic synthesis. Geochim. Cosmochim. Acta.

[26] Burton A., Berger E. (2018). Insights into abiotically-generated amino acid enantiomeric excesses found in meteorites. Life.

[27] Degens E.T., Bajor M. (1962). Amino acids and sugars in the Bruderheim and Murray meteorite. Naturwissenschaften.

[28] Kaplan I.R., Degens E.T., Reuter J.H. (1963). Organic compounds in stony meteorites. Geochim. Cosmochim. Acta.

[29] Burton A.S., Elsila J.E., Hein J.E., Glavin D.P., Dworkin J.P. (2013). Extraterrestrial amino acids identified in metal-rich CH and CB carbonaceous chondrites from antarctica. Meteorit. Planet. Sci..

[30] Hayes J.M. (1967). Organic constituents of meteorites—A review. Geochim. Cosmochim. Acta.

[31] Cooper G., Rios A.C. (2016). Enantiomer excesses of rare and common sugar derivatives in carbonaceous meteorites. Proc. Natl. Acad. Sci. USA.

[32] Partridge R.D., Weiss A.H., Todd D. (1972). Branched-chain carbohydrate structures resulting from formaldehyde condensation. Carbohydr. Res..

[33] Decker P., Schweer H., Pohlamnn R. (1982). Bioids: X. Identification of formose sugars, presumable prebiotic metabolites, using capillary gas chromatography/gas chromatography—Mass spectrometry of *n*-butoxime trifluoroacetates on OV-225. J. Chromatogr. A.

[34] Kawasaki T., Hatase K., Fujii Y., Jo K., Soai K., Pizzarello S. (2006). The distribution of chiral asymmetry in meteorites: An investigation using asymmetric autocatalytic chiral sensors. Geochim. Cosmochim. Acta.

[35] Cleaves H.J., Egel R., Lankenau D.-H., Mulkidjanian A.Y. (2011). A hypothesis for a unified mechanism of formation and enantioenrichment of polyols and aldaric, aldonic, amino, hydroxy and sugar acids in carbonaceous chondrites. Origins of Life: The Primal Self-Organization.

[36] Mizuno T., Weiss A.H., Tipson R.S., Horton D. (1974). Synthesis and utilization of formose sugars. Advances in Carbohydrate Chemistry and Biochemistry.

[37] Meinert C., Myrgorodska I., de Marcellus P., Buhse T., Nahon L., Hoffmann S.V., d’Hendecourt L.L.S., Meierhenrich U.J. (2016). Ribose and related sugars from ultraviolet irradiation of interstellar ice analogs. Science.

[38] Delpoux O., Gourier D., Vezin H., Binet L., Derenne S., Robert F. (2011). Biradical character of D-rich carriers in the insoluble organic matter of carbonaceous chondrites: A relic of the protoplanetary disk chemistry. Geochim. Cosmochim. Acta.

[39] Butlerow A.M. (1861). Formation synthétique d’une substance sucrée. Compt. Rendus Acad. Sci..

[40] Walker J.F. (1964). Formaldehyde.

[41] Delidovich I.V., Simonov A.N., Taran O.P., Parmon V.N. (2014). Catalytic formation of monosaccharides: From the formose reaction towards selective synthesis. ChemSusChem.

[42] Socha R.F., Weiss A.H., Sakharov M.M. (1980). Autocatalysis in the formose reaction. React. Kinet. Catal. Lett..

[43] Hollis J.M., Lovas F.J., Jewell P.R. (2000). Interstellar glycolaldehyde: The first sugar. Astrophys. J..

[44] Milam S.N., Remijan A.J., Womack M., Abrell L., Ziurys L.M., Wyckoff S., Apponi A.J., Friedel D.N., Snyder L.E., Veal J.M. (2006). Formaldehyde in comets C/1995 O1 (Hale-Bopp), C/2002 T7 (LINEAR), and C/2001 Q4 (NEAT): Investigating the cometary origin of H_2_CO. Astrophys. J..

[45] List of Interstellar and Circumstellar Molecules. https://www.astro.uni-koeln.de/cdms/molecules.

[46] Lerner N.R., Cooper G.W. (2005). Iminodicarboxylic acids in the murchison meteorite: Evidence of strecker reactions. Geochim. Cosmochim. Acta.

[47] Pizzarello S., Schrader D.L., Monroe A.A., Lauretta D.S. (2012). Large enantiomeric excesses in primitive meteorites and the diverse effects of water in cosmochemical evolution. Proc. Natl. Acad. Sci. USA.

[48] Brearley A., Hutcheon I., Browning L. Compositional zoning and Mn-Cr systematics in carbonates from the Y791198 CM2 carbonaceous chondrite. Proceedings of the 32nd Lunar and Planetary Science XXXII.

[49] De Leuw S., Rubin A.E., Wasson J.T. (2010). Carbonates in cm chondrites: Complex formational histories and comparison to carbonates in CI chondrites. Meteorit. Planet. Sci..

[50] Lee M.R., Lindgren P., Sofe M.R. (2014). Aragonite, breunnerite, calcite and dolomite in the CM carbonaceous chondrites: High fidelity recorders of progressive parent body aqueous alteration. Geochim. Cosmochim. Acta.

[51] Brearley A.J., Lauretta D., Leshin L.A., McSween H.Y. (2006). The action of water. Meteorites and the Early Solar System II.

[52] Guo W., Eiler J.M. (2007). Temperatures of aqueous alteration and evidence for methane generation on the parent bodies of the CM chondrites. Geochim. Cosmochim. Acta.

[53] Fujiya W. (2018). Oxygen isotopic ratios of primordial water in carbonaceous chondrites. Earth Planet. Sci. Lett..

[54] Harju E.R., Rubin A.E., Ahn I., Choi B.-G., Ziegler K., Wasson J.T. (2014). Progressive aqueous alteration of CR carbonaceous chondrites. Geochim. Cosmochim. Acta.

[55] Bose M., Floss C., Stadermann F.J., Stroud R.M., Speck A.K. (2012). Circumstellar and interstellar material in the CO3 chondrite ALHA77307: An isotopic and elemental investigation. Geochim. Cosmochim. Acta.

[56] Agarwal V.K., Schutte W., Greenberg J.M., Ferris J.P., Briggs R., Connor S., Vandebult C.P.E.M., Baas F. (1985). Photochemical-reactions in interstellar grains photolysis of CO, NH_3_, and H_2_O. Orig. Life Evol. Biosph..

[57] Bernstein M.P., Dworkin J.P., Sandford S.A., Cooper G.W., Allamandola L.J. (2002). Racemic amino acids from the ultraviolet photolysis of interstellar ice analogues. Nature.

[58] Muñoz Caro G.M., Meierhenrich U.J., Schutte W.A., Barbier B., Segovia A.A., Rosenbauer H., Thiemann W.H.P., Brack A., Greenberg J.M. (2002). Amino acids from ultraviolet irradiation of interstellar ice analogues. Nature.

[59] Nuevo M., Auger G., Blanot D., d’Hendecourt L. (2008). A detailed study of the amino acids produced from the vacuum UV irradiation of interstellar ice analogs. Orig. Life Evol. Biosph..

[60] Nuevo M., Materese C.K., Sandford S.A. (2014). The photochemistry of pyrimidine in realistic astrophysical ices and the production of nucleobases. Astrophys. J..

[61] Materese C.K., Nuevo M., Sandford S.A. (2017). The formation of nucleobases from the ultraviolet photoirradiation of purine in simple astrophysical ice analogues. Astrobiology.

[62] De Marcellus P., Bertrand M., Nuevo M., Westall F., Le Sergeant d’Hendecourt L. (2011). Prebiotic significance of extraterrestrial ice photochemistry: Detection of hydantoin in organic residues. Astrobiology.

[63] De Marcellus P., Meinert C., Myrgorodska I., Nahon L., Buhse T., Le Sergeant d’Hendecourt L., Meierhenrich U.J. (2015). Aldehydes and sugars from evolved precometary ice analogs: Importance of ices in astrochemical and prebiotic evolution. Proc. Natl. Acad. Sci. USA.

[64] McDonald G.D., Whited L.J., DeRuiter C., Khare B.N., Patnaik A., Sagan C. (1996). Production and chemical analysis of cometary ice tholins. Icarus.

[65] Nuevo M., Bredehoft J.H., Meierhenrich U.J., d’Hendecourt L., Thiemann W.H.P. (2010). Urea, glycolic acid, and glycerol in an organic residue produced by ultraviolet irradiation of interstellar/pre-cometary ice analogs. Astrobiology.

[66] Kaiser R.I., Maity S., Jones B.M. (2015). Synthesis of prebiotic glycerol in interstellar ices. Angew. Chem. Int. Ed..

[67] Henderson B.L., Gudipati M.S. (2015). Direct detection of complex organic products in ultraviolet (Lyα) and electron-irradiated astrophysical and cometary ice analogs using two-step laser ablation and ionization mass spectrometry. Astrophys. J..

[68] Maity S., Kaiser R.I., Jones B.M. (2015). Formation of complex organic molecules in methanol and methanol–carbon monoxide ices exposed to ionizing radiation—A combined FTIR and reflectron time-of-flight mass spectrometry study. Phys. Chem. Chem. Phys..

[69] Fedoseev G., Chuang K.J., Ioppolo S., Qasim D., van Dishoeck E.F., Linnartz H. (2017). Formation of glycerol through hydrogenation of CO ice under prestellar core conditions. Astrophys. J..

[70] Nuevo M., Cooper G., Sanders J.M., Buffo C.E., Materese C.K., Sandford S.A. Detailed study of the formation of sugar derivatives produced from the uv irradiation of astrophysical ice analogs. Proceedings of the American Chemical Society Spring Meeting.

[71] Cooper G.W., Thiemens M.H., Jackson T., Chang S. (1997). Sulfur and hydrogen isotope anomalies in meteoritic sulfonic acids. Science.

[72] Thiemens M.H., Shaheen R. (2014). Mass-independent isotopic composition of terrestrial and extraterrestrial materials. Treatise Geochem..

[73] Kopetzki D., Antonietti M. (2011). Hydrothermal formose reaction. New J. Chem..

[74] De Bruijn J.M., Kieboom A.P.G., Van Bekkum H. (1986). Reactions of monosaccharides in aqueous alkaline solutions. Sugar Technol. Rev..

[75] Lambert J.B., Gurusamy-Thangavelu S.A., Ma K. (2010). The Silicate-Mediated formose reaction: Bottom-up synthesis of sugar silicates. Science.

[76] Pizzarello S., Cronin J.R. (2000). Non-racemic amino acids in the Murray and Murchison meteorites. Geochim. Cosmochim. Acta.

[77] Goesmann F., Rosenbauer H., Bredehöft J.H., Cabane M., Ehrenfreund P., Gautier T., Giri C., Krüger H., Le Roy L., MacDermott A.J. (2015). Organic compounds on comet 67P/Churyumov-Gerasimenko revealed by COSAC mass spectrometry. Science.

[78] Wright I.P., Sheridan S., Barber S.J., Morgan G.H., Andrews D.J., Morse A.D. (2015). Cho-bearing organic compounds at the surface of 67P/Churyumov-Gerasimenko revealed by Ptolemy. Science.

[79] Altwegg K., Balsiger H., Berthelier J.J., Bieler A., Calmonte U., Fuselier S.A., Goesmann F., Gasc S., Gombosi T.I., Le Roy L. (2017). Organics in comet 67P—A first comparative analysis of mass spectra from ROSINA–DFMS, cosac and ptolemy. Mon. Not. R. Astron. Soc..

[80] Reinhard R. (1986). The Giotto encounter with comet Halley. Nature.

[81] Huebner W.F., Boice D.C., Sharp C.M. (1987). Polyoxymethylene in Comet Halley. Astrophys. J..

[82] Butscher T., Duvernay F., Danger G., Chiavassa T. (2016). Radical-induced chemistry from VUV photolysis of interstellar ice analogues containing formaldehyde. Astron. Asrophys..

[83] Yanlong G., Joël B., François J. (2008). Glycerol as an efficient promoting medium for organic reactions. Adv. Synth. Catal..

[84] Chyba C., Sagan C. (1992). Endogenous production, exogenous delivery and impact-shock synthesis of organic molecules: An inventory for the origins of life. Nature.

[85] Rikken G.L.J.A., Raupach E. (2000). Enantioselective magnetochiral photochemistry. Nature.

[86] Kleindienst P., Wagnière G.H. (1998). Interferometric detection of magnetochiral birefringence. Chem. Phys. Lett..

[87] Banerjee-Ghosh K., Ben Dor O., Tassinari F., Capua E., Yochelis S., Capua A., Yang S.-H., Parkin S.S.P., Sarkar S., Kronik L. (2018). Separation of enantiomers by their enantiospecific interaction with achiral magnetic substrates. Science.

[88] Flores J.J., Bonner W.A., Massey G.A. (1977). Asymmetric photolysis of (rs)-leucine with circularly polarized UV light. J. Am. Chem. Soc..

[89] Takano Y., Takahashi J., Kaneko T., Marumo K., Kobayashi K. (2007). Asymmetric synthesis of amino acid precursors in interstellar complex organics by circularly polarized light. Earth Planet. Sci. Lett..

[90] Nuevo M., Meierhenrich U.J., Muñoz Caro G.M., Dartois E., d’Hendecourt L., Deboffle D., Auger G., Blanot D., Bredehöft J.H., Nahon L. (2006). The effects of circularly polarized light on amino acid enantiomers produced by the UV irradiation of interstellar ice analogs. Astron. Astrophys..

[91] De Marcellus P., Meinert C., Nuevo M., Filippi J.-J., Danger G., Deboffle D., Nahon L., Le Sergeant d’Hendecourt L., Meierhenrich U.J. (2011). Non-racemic amino acid production by UV irradiation of achiral interstellar ice analogs with circularly polarized light. Astrophys. J. Lett..

[92] Modica P., Meinert C., de Marcellus P., Nahon L., Meierhenrich U.J., d’Hendecourt L.L. (2014). Enantiomeric excesses induced in amino acids by ultraviolet circularly polarized light irradiation of extraterrestrial ice analogs: A possible source of asymmetry for prebiotic chemistry. Astrophys. J..

[93] Lucas P.W., Hough J.H., Bailey J., Chrysostomou A., Gledhill T.M., McCall A. (2005). UV circular polarisation in star formation regions: The origin of homochirality?. Orig. Life Evol. Biosph..

[94] Chuang K.J., Fedoseev G., Qasim D., Ioppolo S., van Dishoeck E.F., Linnartz H. (2017). Production of complex organic molecules: H-atom addition versus UV irradiation. Mon. Not. R. Astron. Soc..

[95] Glavin D.P., Dworkin J.P. (2009). Enrichment of the amino acid L-isovaline by aqueous alteration on CI and CM meteorite parent bodies. Proc. Natl. Acad. Sci. USA.

[96] Martins Z., Modica P., Zanda B., Le Sergeant d’Hendecourt L. (2015). The amino acid and hydrocarbon contents of the Paris meteorite: Insights into the most primitive CM chondrite. Meteorit. Planet. Sci..

[97] Pizzarello S., Huang Y., Alexandre M.R. (2008). Molecular asymmetry in extraterrestrial chemistry: Insights from a pristine meteorite. Proc. Natl. Acad. Sci. USA.

[98] Peltzer E.T., Bada J.L., Schlesinger G., Miller S.L. (1984). The chemical conditions on the parent body of the murchison meteorite: Some conclusions based on amino, hydroxy and dicarboxylic acids. Adv. Space Res..

[99] Cronin J.R., Pizzarello S. (1997). Enantiomeric excesses in meteoritic amino acids. Science.

[100] Nuevo M., Meierhenrich U.J., d’Hendecourt L., Muñoz Caro G.M., Dartois E., Deboffle D., Thiemann W.H.P., Bredehöft J.H., Nahon L. (2007). Enantiomeric separation of complex organic molecules produced from irradiation of interstellar/circumstellar ice analogs. Adv. Space Res..

[101] Nuevo M., Chen Y.J., Yih T.S., Ip W.H., Fung H.S., Cheng C.Y., Tsai H.R., Wu C.Y.R. (2007). Amino acids formed from the uv/euv irradiation of inorganic ices of astrophysical interest. Adv. Space Res..

[102] Throop H.B. (2011). UV photolysis, organic molecules in young disks, and the origin of meteoritic amino acids. Icarus.

[103] Ciesla F.J., Sandford S.A. (2012). Organic synthesis via irradiation and warming of ice grains in the solar nebula. Science.

[104] Rodgers C.T. (2009). Magnetic field effects in chemical systems. Pure Appl. Chem..

[105] Adams F.C., Gregory S.G. (2012). Magnetically controlled accretion flows onto young stellar objects. Astrophys. J..

[106] Pizzarello S., Weber A.L. (2004). Prebiotic amino acids as asymmetric catalysts. Science.

[107] Carey F.A., Sundberg R. (2000). Advanced Organic Chemistry, Part A: Structure and Mechanisms.

[108] Martins Z., Alexander C.M., Orzechowska G.E., Fogel M.L., Ehrenfreund P. (2007). Indigenous amino acids in primitive cr meteorites. Meteorit. Planet. Sci..

